# Silk and its composites for humidity and gas sensing applications

**DOI:** 10.3389/fchem.2023.1141259

**Published:** 2023-03-20

**Authors:** Shubhanth Jain, V. Vedavyas, R. V. Prajwal, Malavika Shaji, Vishnu G Nath, S. Angappane, Govindaraj Achutharao

**Affiliations:** ^1^ Solid State and Structural Chemistry Unit, Indian Institute of Science, Bengaluru, India; ^2^ Centre for Nano Science and Engineering, Indian Institute of Science, Bengaluru, India; ^3^ Centre for Nano and Soft Matter Sciences, Bengaluru, India

**Keywords:** silk, fibroins, sericin, degumming, sensors, gas sensing, humidity sensing

## Abstract

Silk fibroin (SF) is a natural protein largely used in the textile industry with applications in bio-medicine, catalysis as well as in sensing materials. SF is a fiber material which is bio-compatible, biodegradable, and possesses high tensile strength. The incorporation of nanosized particles into SF allows the development of a variety of composites with tailored properties and functions. Silk and its composites are being explored for a wide range of sensing applications like strain, proximity, humidity, glucose, pH and hazardous/toxic gases. Most studies aim at improving the mechanical strength of SF by preparing hybrids with metal-based nanoparticles, polymers and 2D materials. Studies have been conducted by introducing semiconducting metal oxides into SF to tailor its properties like conductivity for use as a gas sensing material, where SF acts as a conductive path as well as a substrate for the incorporated nanoparticles. We have reviewed gas and humidity sensing properties of silk, silk with 0D (i.e., metal oxide), 2D (e.g., graphene, MXenes) composites. The nanostructured metal oxides are generally used in sensing applications, which use its semiconducting properties to show variation in the measured properties (e.g., resistivity, impedance) due to analyte gas adsorption on its surface. For example, vanadium oxides (i.e., V_2_O_5_) have been shown as candidates for sensing nitrogen containing gases and doped vanadium oxides for sensing CO gas. In this review article we provide latest and important results in the gas and humidity sensing of SF and its composites.

## 1 Introduction

Sensors are devices that produce a response to any change in the various environmental stimuli, like touch, light, pressure, temperature, presence of gases, to name a few. These sensors are indispensable components of all the instruments/gadgets around us. Our handheld smartphones have a collection of sensors, ranging from capacitive touch sensors to proximity sensors to light sensors. The elevators use touch-sensitive tactile sensors for input. Further, the self-driving car is one of the major innovations currently, and it relies on the input from a horde of sensors like the infrared light sensor to plot a 3D view of the surrounding, radio wave sensor to determine the surrounding objects’ positioning, and velocity, sonar sound sensors to resolve the distance of objects, among many others. The sensors are mainly manufactured with metal oxide semiconductors, although various other materials, like polymers and biomaterials, are being tested nowadays. Based on the input parameter, we have different classes of sensors. Temperature sensors produce a variation in the potential difference between two metals, which is measured across the diode in response to changes in environmental temperature. Proximity sensors indicate the presence of objects nearby without contact. Touch sensors produce an electric signal due to the closure of the electric circuit on contact. The color sensor checks the wavelength of input light and produces an output to match it. Gas sensors display a change in the resistance or the capacitance, conditional on the presence of target gases. A sub-category in this, the humidity sensor monitors the concentration of water vapor in the surroundings. Most commercial humidity sensors also contain a temperature sensor since they measure relative humidity, which is dependent on the temperature. Another sub-category is the alcohol sensor that is used by law enforcement to check the alcohol level in the system. Generally, the response could be shown in an analog meter, such as electric meters, thermometers, or vacuum gauges, or the response could be on a digital display, like most of the modern sensors (e.g., temperature controllers, digital accelerometers, proximity sensors, light sensors or gas sensors). These are a few important sensors available, and in this review, we have focused on gas sensors and humidity sensors.

In order to facilitate a cleaner and better environment for a healthier future, the use of dangerous substances, toxic and combustible gases and pollutants are to be monitored. The detection techniques which are used include: spectrophotometry (Bricker and Johnson, 1945), chromatographic analysis (Beasley et al., 1980), electrochemical analysis (Herschkovitz et al., 2000), catalytic luminescence analysis (Zhou et al., 2006) and gas sensors (Zhang Y. M. et al., 2014). Quick detection with high accuracy is the advantage of the first four techniques mentioned, while portability, cost of measurement, costly instrumentation, and large volume hinder its extensive widespread usage. Therefore, gas sensors which have high sensitivity and facile operation, are used as an alternative technique to detect these dangerous pollutants. Gas sensors are miniature devices available at low cost, suitable for identifying and real-time monitoring of gases suitable for indoor and outdoor applications with high sensitivity and facile operation. The working principle of these gas sensors is based on changes in properties like conductivity, capacitance, impedance, mass, and refractive index of gas-sensing material after interacting (adsorption or absorption) with analyte gases, which is a proportional measure of the concentration of the gases. A strong influence of structure, grain size, morphology, and surface area on the quantity of adsorbed analyte molecules is well known (Song et al., 2012; Wang et al., 2012; Kalantar-zadeh et al., 2016; Gao R. et al., 2018; Ding et al., 2018).

Human activity in the name of evolution and modernization is affecting the ecosystem/nature adversely. To mitigate this, the use of biocompatible or biodegradable materials is stressed in all fields of research. Two ways to approach this are (a) synthetic production of such materials in the laboratory or (b) modifying naturally available materials. Synthetic production requires extensive research on the procedure for preparations and control over the possible harmful by-products during the synthesis, while the naturally available sources need to be modified to some extent for fine-tuning of the properties to overcome drawbacks. Both approaches are being explored by researchers (Ammala et al., 2011). Biomaterials are increasing the quality of electronic devices and paving roads to integrating them deeper into our lives. Some materials, like silk, cellulose, and chitin have been inspected more than others, with each having its advantages and disadvantages (Badawy et al., 2021). Among them, silk is a highly desired material to produce owing to its mass production capabilities with applicability in bio-friendly flexible electronic devices (Gupta et al., 2010; Hu et al., 2012a; Gao R. et al., 2018), due to its excellent mechanical properties, optical properties, and in biomedicine (Wenk et al., 2011; Tsioris et al., 2012; DeMuth et al., 2014; Di Buduo et al., 2015; Holland et al., 2019), biophotonics (Shimanovich et al., 2018) due to biodegradability, biocompatibility, and implantability (Vollrath and Knight, 2001; Du et al., 2006; Jiang et al., 2007; Gupta et al., 2010).

Silk is being explored in diverse fields. To state a few are as follows. Jung et al. (2014) used soft lithographic patterning to fabricate graphene-based flexible devices using surface energy modification for high-performance graphene-based flexible devices such as transistors, chemical sensors, and devices with SF as substrate. Xie’s group fabricated nacre-mimetic nano-hybrid, which provides hybridized dynamic feedback using alternating diverse mechanical properties of nanoscale graphitic oxide (GO_x_) and SF materials composites (Xie et al., 2018).

Reizabal et al. (2022) showed SF nanohybrids with 10 wt% Indium Tin Oxide (ITO) can be used for capacitive touch sensing applications. Abadi et al. (2021) developed excellent piezoresistive pressure-sensing devices as well as sorbents for oil/water separation with electrically conductive and mechanically flexible MXene-SF composite foams, which were nacre-mimetic. For the application in hydrogen peroxide identification from a non-enzymatic electrochemical method, Au-ZnO nanocomposites with SF template were shown by Chen and coworkers (Chen et al., 2018).

Li et al. (2012) fabricated disposable amperometric *α*-fetoprotein (68 kDa) electrochemical immuno-sensor films using SF protein membrane, Prussian blue, and gold nanoparticles. Genovese et al. (2017) prepared light-responsive silk nanofiber composites by doping photo-chromic spiropyran to silk fibroin poly(ethylene oxide) nanofibers.

Sericulture (i.e., the cultivation of silkworms) is carried out in many countries, and India is the second largest silk producer, with China claiming the top spot (Anitha, 2011). India produced a total of 33,739 metric tons (MT) of raw silk in 2020–2021, having mainly four varieties, Muga, Eri, Mulberry, and Tusar (Government of India, 2023).

## 2 Silk structure and processing

### 2.1 Structure

**FIGURE 1:**
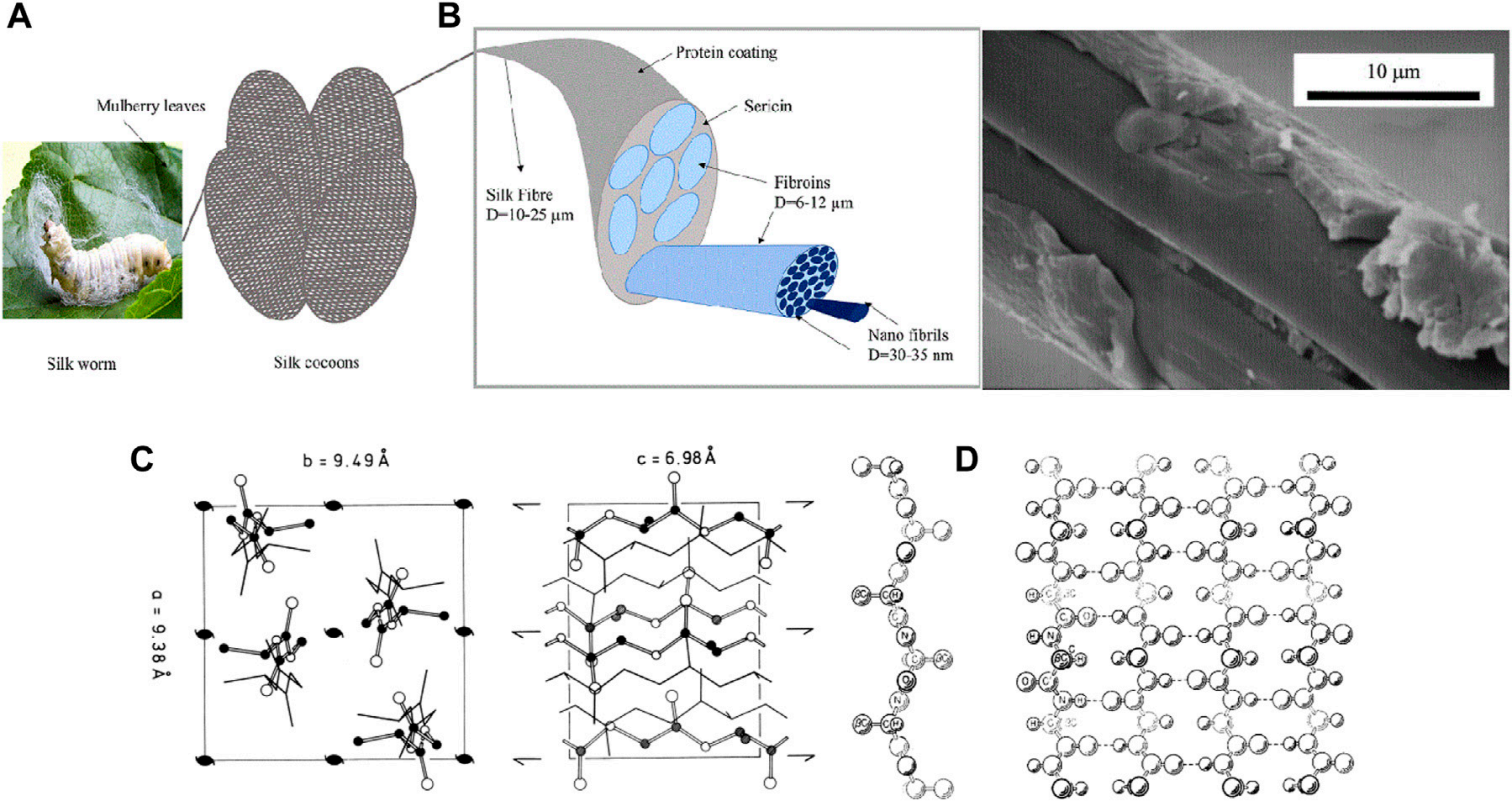
**(A)** Silkworm feeding on mulberry leaves and formation of cocoon **(B)** SEM image showing structure of sericin on partially degummed silk Reprinted with permission from (Pérez-Rigueiro et al., 2002). Copyright 2002 John Wiley and Sons **(C)** Structure of silk; left—amorphous domains, right—*β*-sheet crystallites Reprinted with permission from (Takahashi et al., 1999). Copyright 1999 Elsevier **(D)** Alignment of silk in *β*-sheet structure showing the inter-unit H bond Reprinted with permission from (Marsh et al., 1955). Copyright 1955 Elsevier.

Raw silk is obtained from silkworms. Silkworms consume mulberry leaves for 20–35 days, increasing their size by about 3–5 inches. While forming a cocoon, they start moving their bodies in the shape of the number eight around 3 × 10^5^ times, which takes 3–8 days to form a cocoon. In Figure 1A, we have shown the silkworm feeding on mulberry leaves, the formation of a cocoon, and the structure of silk fibers. Each silk cocoon consists of strands of fibroins which are 100 m long fibers, clung together by a protein gum called sericin (Mondal et al., 2007; Babu, 2020). The cocoon consists of 70%–80% silk fibroins (SF) which are hydrophobic, 20%–30% of hydrophilic sericin, 0.4%–0.8% of wax, and 0.2% natural colour.

Sericin is a water-soluble globular protein made of side chains containing polar entities like amino, hydroxyl, and carboxyl functional groups. It is present as a tube-like enclosure over the silk fibroin as shown in SEM Figure 1B (Pérez-Rigueiro et al., 2002), with molecular weight distribution varying between 10 and 400 kDa (Matsuhira and Osaki, 2015; Eshchanov et al., 2021). Sericin has exclusive functionalities due to its molecular structure. Properties like hygroscopy and biological activities, which include tyrosinase activity inhibition, antioxidation, and anticancer activity, are seen in sericin (Kato et al., 1998; Aramwit and Sangcakul, 2007). It is a readily available bio-compatible renewable resource.

SF is made of amorphous domains and *β*-sheet crystallites. The presence of hydrogen bonds between adjacent peptide blocks or in a single block gives the *β*-sheet conformation its crystallite structure as shown in Figure 1C (Takahashi et al., 1999). The linear sequence of amino acids in SF protein influences the dominant *β*-sheet secondary structure. The repetition in the amino acid arrangement in the crystalline domain leads to inter-unit hydrogen bonds and causes the development of a two-dimensional folded chain, which is aligned anti-parallel to the *β*-sheet conformation. This is seen in Figure 1D (Marsh et al., 1955). 3D nano-crystals are formed due to van der Waals interactions between stacked sheets facilitated by small side chains of amino acids like glycine and aniline (Hardy et al., 2008). SFs are composed of *β*-sheet crystallite, which is a rectangular lattice. Its 3D structure is defined with amino acid side chains along the *x*-axis, hydrogen bonds along the *y*-axis, and peptide bonds along the *z*-axis. The calculated lattice parameters are a = 0.938 nm, b = 0.949 nm, and c = 0.698 nm (Karakutuk et al., 2012). SF acts as a competent and low-cost substrate due to the availability of multiple electron offering groups on its surface where metallic ions can attach (Kundu et al., 2008).

SFs are a good candidate for flexible miniature sensor devices due to their tough mechanical nature and compatible fibroin diameter ∼10 *μ*m making them a substrate where active sensing materials can be coated (Hu et al., 2012b; Sheng et al., 2017; Shi et al., 2018; Won et al., 2020). Also, functional groups like amide and hydroxyl help in binding polymers and organic moieties through hydrogen bonding. SF is a highly ordered semi-crystalline polypeptide fiber with a refractive index of ∼1.54, mechanical characteristic with toughness value of 70–78 MJ/m^3^ and Young’s modulus of 15–17 GPa (Gosline et al., 1999; Pérez-Rigueiro et al., 2000), with considerable moisture affinity. So SFs mainly act as substrate materials with good tensile strength, surface area, and ease of processing with different materials.

### 2.2 Processing of silk

Degumming is a process of removing sericin from silk. Various techniques are employed in degumming, such as extracting with water, boiling off in soap, using alkali/organic acids or amines, or by ultrasonication (Gulrajani, 1992; Sothornvit et al., 2010; Choudhury and Devi, 2016; Oduor et al., 2020). The water extraction method is preferred since it is cost-friendly and chemical-free. The hydrothermal method of degumming takes the least amount of time. The effect of temperature on the degumming ratio is listed in Table 1. A consistent temperature should be maintained for the hydrothermal treatment to ensure a constant degumming ratio. Later, the fibroins are rinsed with DI water and collected. The SFs will have a rough texture if the degumming ratio drops below 26%, and it gains a yellowish hue if the temperature exceeds 120°C. Sericin can be re-extracted from the solution using dialysis.

**TABLE 1:** Details of effect of temperature and water ratio on silk degumming in hydrothermal method.

Sl.No	Ratio (silk:water) w/w	Temperature (°C)	Degumming ratio (%)
1	1:40	120	∼26
2	1:40	110	∼23
3	1:40	100–105	∼14–16
4	1:40	150–160	∼29
5	1:40	130–140	∼23–24
6	1:50	130–140	∼23–24

Annealing silk in an inert atmosphere introduces structural changes with drastic variations in the properties. The silk proteins are transformed into a carbonaceous solid, called pyroproteins, on heating over 350°C where the *β*-sheet structure of proteins transforms into sp^2^-hybridized carbon in hexagonal structure (Cho et al., 2015). Protein in SF is related to a char-type polymeric precursor that will not melt on annealing. Having no aromatic backbone or cross-linked structures, *β*-sheet-rich proteins of SF undergo restructuring to form a conjugated molecule on pyrolysis.

## 3 Sensing application

**FIGURE 2:**
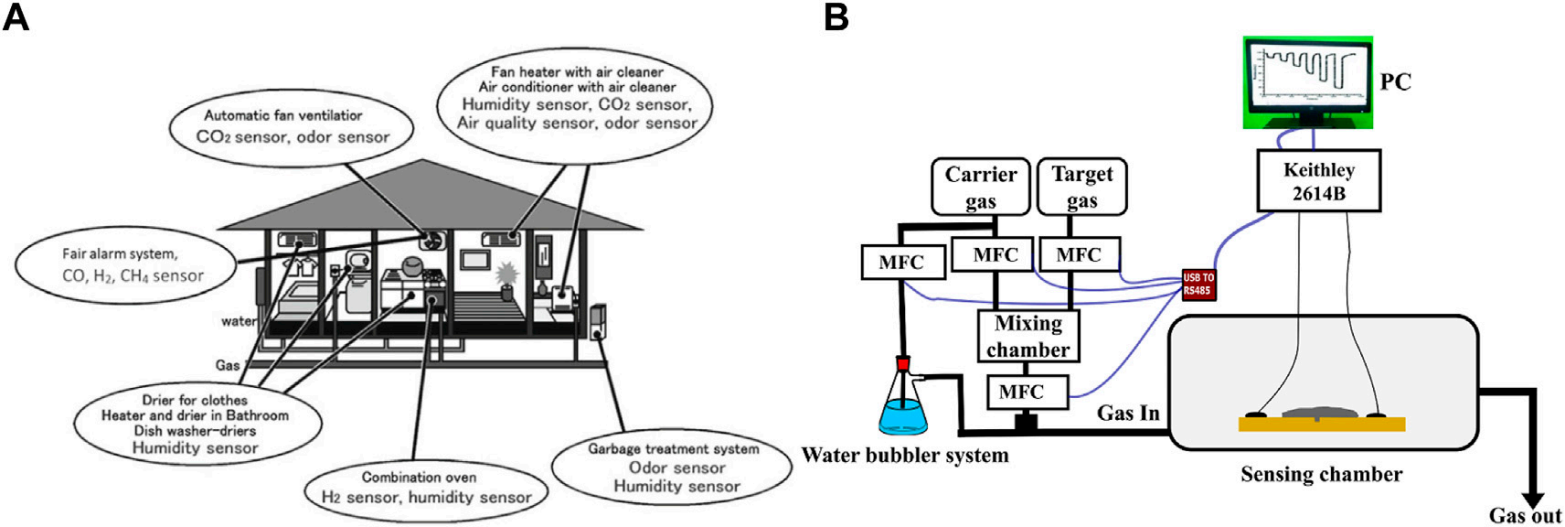
**(A)** Gas sensor variations for use at a modern home (Korotčenkov, 2013) **(B)** Schematic representation of setup used in gas sensing measurements.

Presently, the use and mass production of precarious materials, especially combustible and toxic gases, in industries have become extensive. Any accidental escape of these gases to the atmosphere could be a potential hazard. The concept of gas sensing is thus implemented to avoid such situations. There is extensive use of gas sensors in many areas, including medical institutions, research organizations (environmental and scientific), electronic manufacturing plants, food, and agricultural processing, etc. Gas sensors are becoming widely beneficial, as displayed in Table 2. The use of these gas sensors in households leads to the uplifting of the quality of life of the public, as shown by the cartoon illustration in Figure 2A (Korotčenkov, 2013). Moreover, these devices are also shown to complement the functioning of fire sensors by detecting the presence of gases in smoke and are also being used in electronic noses for quality control in various industries. A typical sensing setup to test materials for gas sensing applications can be seen in Figure 2B.

**TABLE 2:** Examples of gas sensor applications Korotčenkov (2013).

Field of application	Function	Detected gases
Environment	Atmospheric toxic gases due to industrial emissions	CO, CH_4_, humidity, CO_2_, O_3_, NO_x_, volatile organic compounds (VOCs), SO_x_, HCs, NH_3_, H_2_S
Safety at work	Air quality factories	combustible gases, O_2_
Domestic safety	Poisonous gases leak detection and air purifier	Humidity, CO_2_, VOCs, CO, CH_4_
Car safety	Ventilation, gasoline vapour, breath test	CO_2_, O_3_, NO_x_, VOCs, LPG, CH_4_
Public Health	Air quality and Safety checks	Flammable gases, explosives, Toxic gases
Hospitals	Diagnosis (breath analyzer) and drug monitoring	O_2_, NH_3_, NO_x_, CO_2_, H_2_S, H_2_, Cl_2_, anesthesia gas
Agriculture	Flora/fauna diagnostics; water and soil examination; poultry/animal inspection; sewage waste supervision	amines, NH_3_, humidity, CO_2_
Culinary quality	Identification of molecules which are let out by rotting substances	Humidity, CO_2_, NH_3_, etc.
Combustion Engines	Control of the amount of the gases in the engine and gas boiler for efficienct combustion process (in power plants, automotive industry)	O_2_, CO, HCs, NO_x_, SO_x_, CO_2_, H_2_
Industry: Petrochemical, Steel Water treatment, Semiconductor	Quality control; Course monitoring; Workplace monitoring; waste monitoring; leakage alarms	HCs, conventional pollutants, O_2_, H_2_, CO, HCl, Cl_2_, CO_2_, H_2_S, CO, H_2_, AsH_3_, O_3_, H_2_, Cl_2_, TEOS, Si, C_4_F_6_, BCl_3_, C_5_F_8_, HF, GeH_4_, CO, NH_3_, PH_3_, NO_2_
Defense/military	Detection of toxins, biological and chemical warfare agents	Agents, explosives, propellants
Aerospace	Measuring of oxygen and toxic and flammable gases in atmosphere	H_2_, O_2_, CO_2_, humidity
Traffic/tunnels/car parks	Traffic management and control; air quality control in tunnels and car parks	CO, O_3_, NO_x_, SO_2_, CH_4_, LPG

For any sensor fabrication, their sensing parameters, including response/recovery times, flexibility, reproducibility, selectivity, and long-term stability, are investigated. For a certain amount of given gas, the time taken to reach the steady state corresponds to the response time of the sensor. Many articles report response time being the time required for the signal value to get to 90% of its response value (*R*_g_) in the test gas. Recovery time is the time needed for getting back the signal values to 90% of its baseline value (*R*_a_). It is to be noted that in many cases, after each test, the initial signal may not maintain its original value, and the sensor response is calculated using the function of Response% shown in Eq. 1.

$$Response\%=\frac{\left|R_{g}-R_{a}\right|}{R_{a}}\;\times 100\%\;$$ (1)

### 3.1 Sensor classification

Based on the operation principles, gas sensors are categorized into gravimetric which includes surface acoustic waves (SAW) and Quartz Crystal Microbalance (QCM), resistive, capacitive, semiconductor and optical.

#### 3.1.1 Gravimetric sensors

In gravimetric sensors, the variation in frequency due to change in mass caused by adsorption of analyte molecule gives the measurement of analyte gas concentration (Korotčenkov, 2013). The sensitivity of the SAW apparatus to minute variations in surface mass (100 pg/cm^2^) acts as the principle of these gas sensors. The chemical selectivity of these sensors suffers a blow due to the fractional monolayer mass sensitivity. A response is observed irrespective of the moiety adsorbed. One of the ways to overcome this is by using a pair of SAW devices, where the surface of one is altered by a chemically particular interface material based on the interested analyte(s), while the second is chemically passivated or left uncoated. This leads to a similar response by both universally, while the altered material contributes added specific response to the interested analyte(s) (Hoyt et al., 1998). In Quartz Crystal Microbalance (QCM) devices, the change in surface mass (in ng) due to the interaction of sensor material with selective gases causes a change in the frequency of oscillating quartz crystals. The mass of the analyte adsorbed can be determined based on a change of frequency using the Sauerbrey Eq. 2,

△m=−C×△f (2)

**FIGURE 3:**
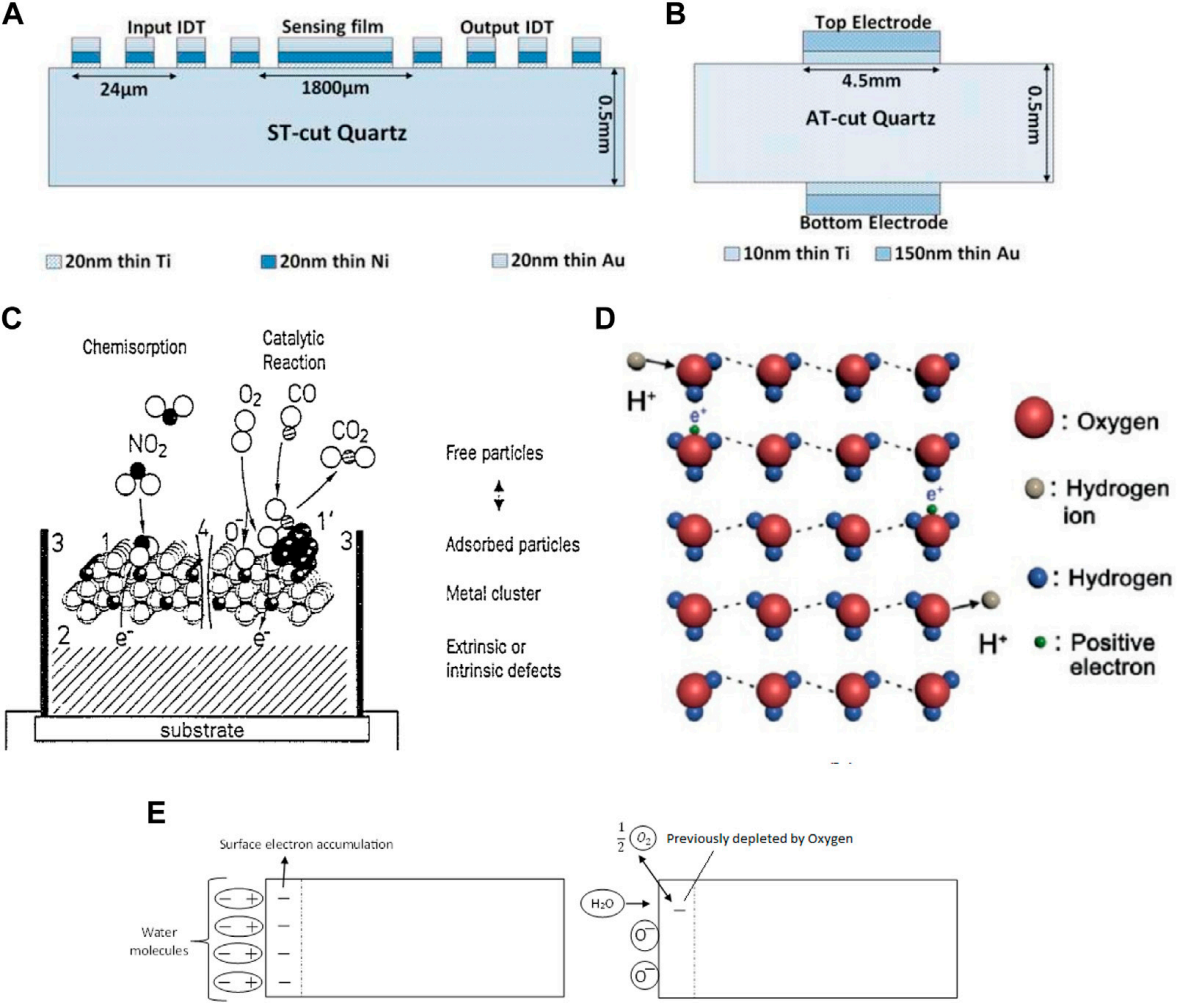
Sensor assembly of **(A)** SAW devices **(B)** QCM devices Reprinted with permission from (Mohibul Kabir et al., 2014) **(C)** The effect of 1) Surface, 2) Bulk, 3) Three-phase boundary, and 4) Grain Boundary towards electrical conduction in a sensor Reprinted with permission from (Göpel and Dieter Schierbaum, 1995). Copyright 1995 Elsevier **(D)** Grotthuss mechanism Reprinted with permission from (Zheng et al., 2021). Copyright 2021 John Wiley and Sons **(E)** Possible mechanism for Resistive humidity sensor Reprinted with permission from (Chen and Lu, 2005). Copyright 2005 American Scientific Publishers.

△*m* represents the change in mass, △*f* is the resonant frequency of the crystal, while C is a constant for a particular sensor, dependent on sensor specifications (Sauerbrey, 1959; Kumar Vashist and Priya, 2011). QCM sensors operate at a 5–20 MHz frequency, while SAW sensors operate at 40–200 MHz. The sensing mechanism is through surface waves in SAW, while in QCM, bulk waves are employed (Mohibul Kabir et al., 2014). The sensing material is coated on one of the surfaces of the crystal in SAW sensors for sensing action, while QCM has electrodes on both sides of the crystal for the purpose as seen in Figures 3A, B respectively (Mohibul Kabir et al., 2014). These differences make SAW sensors a more sensitive type of gravimetric gas sensor.

#### 3.1.2 Resistive sensor

In resistive sensors, changes in the electrical resistance of the sensing layer caused by the interaction with analyte species are usually measured through changes in its resistance or impedance (Korotčenkov, 2013). The changes are dependent on the type of material and the type of analyte, and its concentration. These changes are the consequences of adsorption, chemical reactions, diffusion, catalysis, or swelling on the sensing material due to interactions with a gas analyte. Possible interactions are shown in Figure 3C (Göpel and Dieter Schierbaum, 1995). For example, RH surge usually declines the resistance or impedance of the resistive-type humidity sensor. Grotthuss mechanism is used to explain the typical resistive-type humidity sensor (Chen and Lu, 2005). Molecules of water get chemically adsorbed on an active surface of the sensor, forming a complex that subsequently transforms to surface hydroxyl groups seen in Figure 3D (Zheng et al., 2021). Additional water molecules get adsorbed through hydrogen bonding on the two neighboring hydroxyl groups. The top molecule of condensed water cannot move readily due to the constraint of hydrogen bonding. Thus this physically adsorbed first layer is stagnant, and there are no hydrogen bonds formed between the water molecules in this layer. Hence, no H^+^ conduction at this step. Upon further condensation, additional layers are formed on top of the first physically adsorbed layer, which is barely ordered. As more layers condense, the orderly nature gradually disappears, and protons obtain more and more freedom to navigate inside the condensed water *via* the Grotthuss mechanism. From this, it can be pointed out that pure water-phase protonic conduction is not sensitive at low humidity, at which vapors of water could barely form continuous mobile layers on the sensor material surface. This is referred to as ionic conduction. Conduction through electronic tunneling is attributed to the donation of electrons from the chemically adsorbed water molecules to the surface, which introduces donor surface states near the Fermi level. The surface anions add donor energy levels and serve in conduction. It was proposed that molecules of water substitute the adsorbed ionized oxygen moieties (O^−^, O^2−^, etc.) and thus causing them to release the electrons. Moreover, it was also proposed that due to the polarity of water, the adsorbed water moieties could attract the electrons to the surface, causing an increase in the conductivity. Both mechanism are shown in Figure 3E (Chen and Lu, 2005). This type of sensing is known as the “electronic type” since the conductivity stems from the surface concentration of electrons. The induced energy by the surface anions and the tunneling effect aid surface electron hopping in the immobile layers, thereby assisting in the conductivity.

Although the mainstream resistive gas sensors utilize metal oxides or conductive polymer as sensing materials, various other compounds have been proposed for the fabrication of resistive gas sensors (Neri, 2015). Some of those are:

→ Metal oxides
→ Conducting polymers
→ 2D metal chalcogenides
→ Graphene, CNTs and their derivatives
→ Composite hybrid materials

Of these materials, we have gone into more detail about the working of metal oxide semiconductor.

#### 3.1.2.1 Semiconductor sensor

**FIGURE 4:**
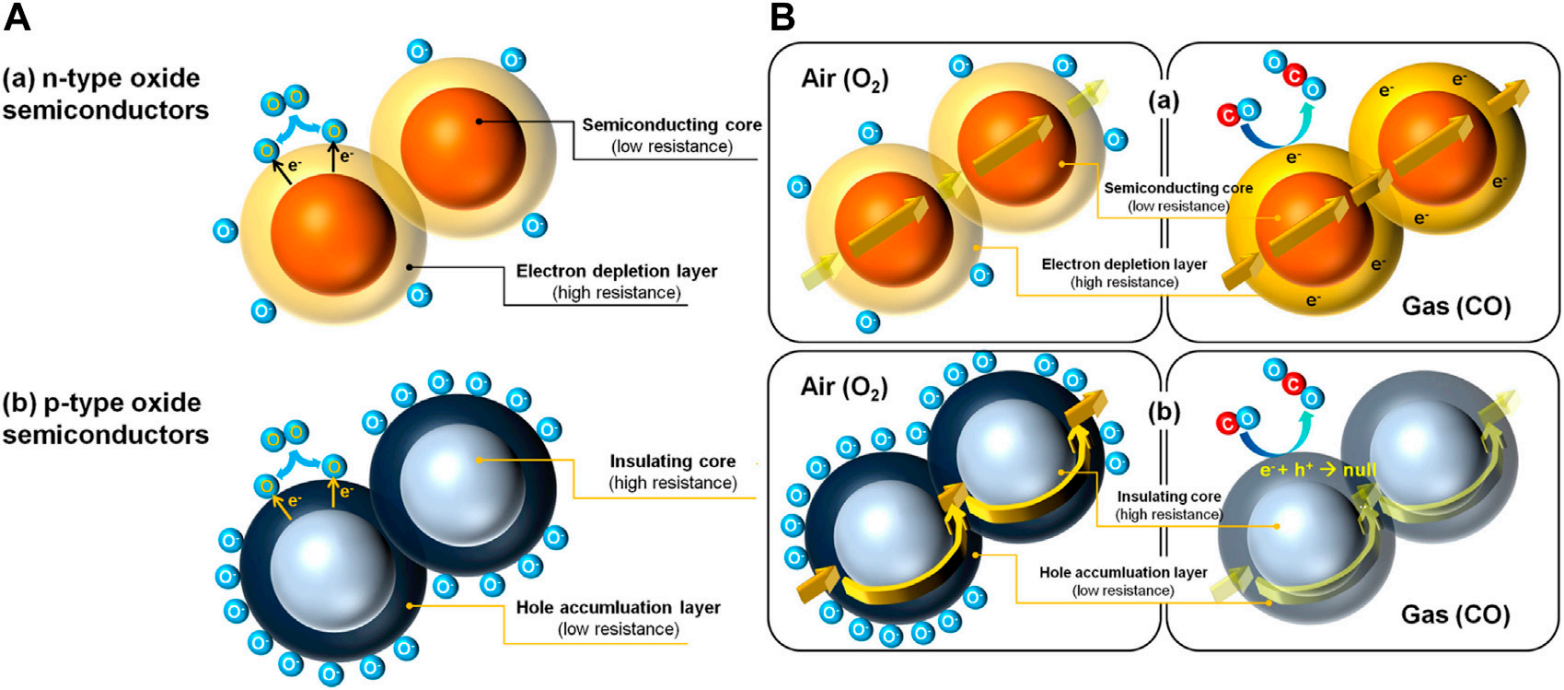
**(A)** Difference in the core of the two types of semiconductors **(B)** Gas sensing in (a) n-type semiconductor and (b) p-type semiconductor Reprinted with permission from (Kim and Lee, 2014). Copyright 2014 Elsevier.

Semiconductor materials are applied in sensor devices at the active materials for detecting reducing gases such as H_2_, CO, VOCs, and other hydrocarbons (Korotčenkov, 2013). This type of semiconductor plays an important role in the sensing application depending on the doping, i.e., p-type and n-type. The distinct sensing mechanism between the two is understood based on the major charge carriers. The surface of the sensors contains adsorbed oxygen shown in Figure 4A, causing a decrease in the electron concentration leading to high resistance in n-type semiconductors (WO_3_, SnO_2_, TiO_2_, In_2_O_3_, ZnO) while instigating a surge in the hole concentration leading to low resistance in p-type semiconductors (Cr_2_O_3_, NiO, CoO, MnO_2_, CuO) (Barsan et al., 2010). This phenomenon is represented in Eq. 3 (Kim and Lee, 2014).

$$O_{2gas}+S_{ads}⇌O_{2ads}+2e^{-}↔2O_{ads}^{-}\;$$ (3)

On an n-type semiconductor surface, ionic oxygen species contribute to the creation of a depletion layer. The depletion layer is the region where an isolated charge has been developed due to electron drain by oxygen species, inhibiting the flow of electrons and leading to increased resistance. The same reaction causes an increase in the concentration of majority charge carriers (holes) in p-type semiconductors, leading to decreased resistance. The presence of reducing gas in the atmosphere produces a counter-reaction shown in Eq. 4.

$$R+O_{ads}^{-}\to RO+S_{ads}+e^{-}\;$$ (4)

Since the electron gets freed up, the resistance is lowered in n-type, which can be envisioned from Figure 4B (a) (Kim and Lee, 2014). This results in a massive response to minor variations in the amounts of different reducing gases in an n-type semiconducting gas sensor as reported (Firooz et al., 2009; Seo et al., 2011; Singh et al., 2011; Zeng et al., 2011; Xu et al., 2013). Whereas, with the release of electrons, the hole population decreases because of electron-hole recombination in a p-type material as illustrated in Figure 4B (b) (Kim and Lee, 2014). The resistance thus increases in response to reducing gases. Hübner et al. (2011) gave the relation between the gas response of p-type (*S*_p_) and n-type (*S*_n_) semiconducting sensors with similar morphologies as given in Eq. 5 (Takahashi et al., 1999).

$$S_{p}=\sqrt{S_{n}}\;$$ (5)

This indicates that although p-type semiconducting sensors are more sensitive, it is very challenging to design a sensor using these materials due to significantly low response% and hence not many p-type gas sensors are available commercially.

These devices also include a heater to maintain a temperature between 200°C and 500°C, depending on the sensor material, for optimum performance. This limits high-temperature sensors’ use in the detection of flammable, explosive, and toxic gas (Tang and Wang, 2015a; Xing et al., 2017; Dey, 2018). As a workaround, UV (*λ* = 365 nm) illumination is being used to lower the operating temperature. Various reports have shown that UV illumination could boost the sensing activity of semi-conducting oxides (Lu et al., 2012; Luís et al., 2017; Meng et al., 2018). Also, UV-photogenerated electrons present on the sample surface increases its conductivity (Gao Z. et al., 2018). Among many kinds of gas sensors, these are mainstream products since neither environmental temperature nor humidity interferes with their working. Semiconductor gas sensor has seen extensive backing for the past 20 years due to their high sensitivity, steady performance, low price, small size, facile use, etc.

#### 3.1.3 Capacitive sensor

Capacitive sensors work on the concept of change in capacitance (Korotčenkov, 2013) due to change in relative dielectric permittivity (*ϵ*_r_), electrode surface area (A), or the separation between the electrodes (d) (Kummer et al., 2004). In regards to the capacitive-type sensor, the variation in the capacitance is attributed to the modification in the relative permittivity of the dielectric medium due to the adsorption or absorption of the molecule of interest into the pores. A surge in the dielectric constant of this system is noticed since most analyte molecules have a large dielectric constant (compared to the dielectric constant of air being 1). The capacitance of the system will vary in accordance with the amount of analyte molecule that has replaced air. In the case of humidity sensors, higher the RH value, higher the capacitance will be due to a higher value of *ϵ*_r_. Capacitive sensors for humidity detection are of two types in general a) sandwich type, where the sensing material is sandwiched between two electrode plates. b) Interdigitated type, where the sensing ceramic layer is placed above the printed interdigitated electrode. Sandwich types typically have fewer parasitic capacitance and higher sensitivity but prolonged response time due to water molecules’ diffusion *via* the electrodes to reach the sensing layer. The interdigital type has a brief response time because of water molecules’ direct reach to the sensing ceramic surface. Also, because of its high intrinsic resistance at low humidity, it has low sensitivity.

### 3.2 Gas sensors

Lightweight, simple to design, and economical and bio-friendly materials are a few of the challenges scientists aspire to meet in the sensor field. Sensing applications like Triboelectric nanogenerators as self-sustaining sensors (i.e., Conversion of mechanical energy from physical movements of the wearer to electrical current through electrostatic induction and coupling effects), optical sensors (i.e., converts light rays into an electronic signal), pressure sensors (i.e., measure pressure and transduce it into an electric signal where the amount is proportional to the pressure applied) have inculcated SF material. Silk-based sensors have been reviewed (Badawy et al., 2021).

#### 3.2.1 NO_x_ (NO, NO_2_)

Nitrogen oxides (NO_x_) are produced during high-temperature fossil-fuels combustions (Katsouyanni, 2003). Nitric oxide (NO) is transformed to nitrogen dioxide (NO_2_) by oxidation, and NO_2_ in reaction with aerosols produce secondary gases. NO_2_ affects the human air tract causing asthma and inhaled allergen response (Bernstein et al., 2004; Kampa and Elias, 2008).

An encouraging viewpoint for graphene-based electronics is the control of the type and density of charge carriers by doping (Zhi and Müllen, 2008; Henry et al., 2011). Adsorption of extrinsic atoms or molecules on the graphene surface produces a doped two-dimensional material. Drastic changes in the magnetic and electronic properties of graphene due to adsorbed species are noticed, helping in the fabrication of extremely sensitive gas sensors (Zhi and Müllen, 2008; Zhang X. et al., 2014; Shim et al., 2015). This is especially seen in the case of exposure to reducing gases. Graphene oxide (GO), a derivative of graphene, can be produced on a large scale using Hummers’ method and has been used for a variety of applications because of the characteristics of the oxygen functional groups (Hummers and Offeman, 1958). Upon removal of some of these through a chemical or thermal reduction process, they can be used for chemo-resistive sensor applications (Won et al., 2021). Based on first-principles calculations, the charge transfer between gas molecules (H_2_O, NH_3_, CO, NO_2_, and NO) and the graphene surface along with energies, position, and orientation of adsorbed gases have been studied (Leenaerts et al., 2008). Adsorbate-induced doping of graphene was investigated using a combination of first-principles calculations, and transport measurements (To et al., 2008). The residual oxygen functional groups produced during the reduction process of GO_x_ provide defective centers, which are active analyte adsorption spots. These adsorption spots boost the interaction of NO_2_ with rGO and offset the gains in sensitivity against the fast decline in conductivity, which improves the sensor activity (Robinson et al., 2008; Dan et al., 2009; JesseFowler et al., 2009).

Won et al. (2020) fabricated electronic textile (e-textile) for the detection of NO_2_ gas using GO_x_ and commercial silk fabric (CSF). The e-textiles were fabricated by soaking the silk fiber into a GO_x_ solution followed by thermal reduction at 400°C without any chemical treatment. On adsorption of NO_2_ gas over reduced graphene oxide (rGO), the former acts as the electron acceptor, while rGO becomes the donor resulting in a hole generation in rGO. This leads to a decline in the electrical resistance of rGO. As NO_2_ interacts with sp^2^-carbon *via* the weak dispersive force with low binding energy, a quick response is observed (Tang and Cao, 2011). On the interaction between oxygen functional groups and NO_2_ gas, a delayed response is seen after the above quick one due to the higher binding energy between them.

It has been previously reported that embedding rGO with ZnO and SnO_2_ enhanced its NO_2_ sensing behavior, but ZnO and SnO_2_ implanted in rGO-CSF e-textiles were found to have reduced gas sensing activity compared to rGO-CSF. The reason given was that the Zn and Sn ions interact with the functional groups of GO_x_ during the synthesis, passivating it. The prepared e-textile annealed at 400°C showed a response to 1 ppm NO_2_ and the best response amongst the rGO-CSFs composites with 24% when it was brought in contact with 10 ppm NO_2_ at ambient temperature and dry conditions.

The highly porous structure with a high surface area of SF makes it a very suitable substrate for the coating of rGO, and its interactions with rGO prompt it as a good sensing device. The textile has also been annealed to 400°C leading to the carbonization of silk (Cho et al., 2015), which results in better conductivity and connectivity due to an increase in the sp^2^-hybridized carbon. This could also result in a better response to the gas.

#### 3.2.2 Hydrogen chloride (HCl)

Hydrogen chloride is a very corrosive acidic gas with a pungent smell that causes severe burns to the skin and serious damage to the eyes on exposure. Inhaling the gas leads to severe burns on the throat and mouth. It reacts with the mucous lining present in the respiratory system and the digestive system leading to possible perforation of the esophagus and stomach. Symptoms of inhalation of HCl gas include cough, shortness of breath, and mucosal irritation while causing respiratory tract damage at a higher concentration. It is also very corrosive/reactive to any metallic objects exposed.

Carbon nanotube (CNT), with its large surface area, is explored as a chemical sensor due to its susceptibility to the chemical environment resulting in variation in conductivity (Frank et al., 1998; Hu et al., 2010; Wang et al., 2017). Conducting polymers like polyaniline (PANI), polypyrrole (PPy), and polythiophene are a few good candidates for sensor applications due to changes in their doping extent, free charge mobility, or the density of free charge carriers on interaction with chemical molecules (Janata and Josowicz, 2003; Zhang et al., 2008).

Sheng et al. (2017) have studied CNT/SF/PANI composites as a sensor for HCl. Composites are prepared using two types of CNTs, i.e., vertically aligned CNT (prepared on a SiO_2_ substrate) and commercially bought random CNT powder. Fibroin solution (prepared by dissolving fibroin threads in CaCl_2_, deionized water, and alcohol) was used to composite with CNT, which led to the formation of microscale porous material. The addition of fibroin solution leads to the direct removal of CNT from the SiO_2_ substrate without any further rigorous acid or sonication treatment. PANI was coated electrochemically (Wang et al., 1987).

Of the two prepared CNT and fibroin composites (CNT-SF), the one with array CNT (CNTA) outperformed with respect to sensitivity, which has been attributed to the high graphitic nature of CNTA. A proportional relationship between the relative sensitivity and the amount of analyte was noticed in CNTA. A distinct response of PANI-functioned CNTA-SF to HCl vapor is seen from CNTA-SF. On exposure to HCl vapors, the resistance of the PANI-functionalized CNT array decreased, while that of pristine CNTA-SF samples increased (with a 400 ppm detection level). The results are tabulated in Table 3.

**TABLE 3:** HCl and NH_3_ sensitivity and detection limit of CNT-SF-PANI composites Adapted with permission from Sheng et al. (2017). Copyright 2017 IOP Publishing.

Vapour	Sample	Relative sensitivity %	Detection limit (ppm)
HCl	CNT powder	0.11	400
	CNTA	0.83	200
	CNTA/PANI (6.7 min)	2.3	200
	CNTA/PANI (20 min)	4.1	200
NH_3_	CNTA	0.69	50
	CNTA/PANI (6.7 min)	1.6	50
	CNTA/PANI (20 min)	2.9	50

The reduced resistance in PANI-functioned CNTA on exposure to HCl vapor is a result of the protonic acid doping activity of PANI, leading to a decline in resistance in PANI (Puthirath et al., 2016). Here the fibroin solution is mainly used to hold the CNTs together and ease their removal from the SiO_2_ substrate.

#### 3.2.3 Ammonia (NH_3_)

The source of Ammonia (NH_3_) gas pollution is attributed to agricultural areas, automobile, fertilizer, pharmaceutical, and synthetic fiber industries. Most significant places are associated with high-density animal farming, and industrial fertilizer production (Van Damme et al., 2018). Excessive inhalation of NH_3_ gas can poison the human body, which leads to pharyngitis, hoarseness of voice, breathing difficulties, and even block trachea (Metin and Metin, 2010). A large amount of NH_3_ in the environment contributes to the acidification and eutrophication of ecosystems (Roland et al., 2010; Paerl et al., 2014). The human detection levels for NH_3_ gases are above 5 ppm, and the recommended exposure limit is 25 ppm.

Pristine and CNTA show high sensitivity for NH_3_ with a detection limit of 50 ppm in comparison with randomly distributed commercially obtained CNTs with a 400 ppm detection level (Sheng et al., 2017). The fabricated CNT/fibroin/PANI composite showed sensitivity to NH_3_ with a detection limit of 50 ppm with 2.9% relative sensitivity. The results are tabulated in Table 3. A better microsensor based on PANI/TiO_2_ on silk fibroin was built by Shi and co-workers to track the freshness of pork by detecting NH_3_ generation. It was reported with a good response time of 10 s for a concentration of 100 ppm of ammonia with a response of 0.82 (Shi et al., 2018).

**FIGURE 5:**
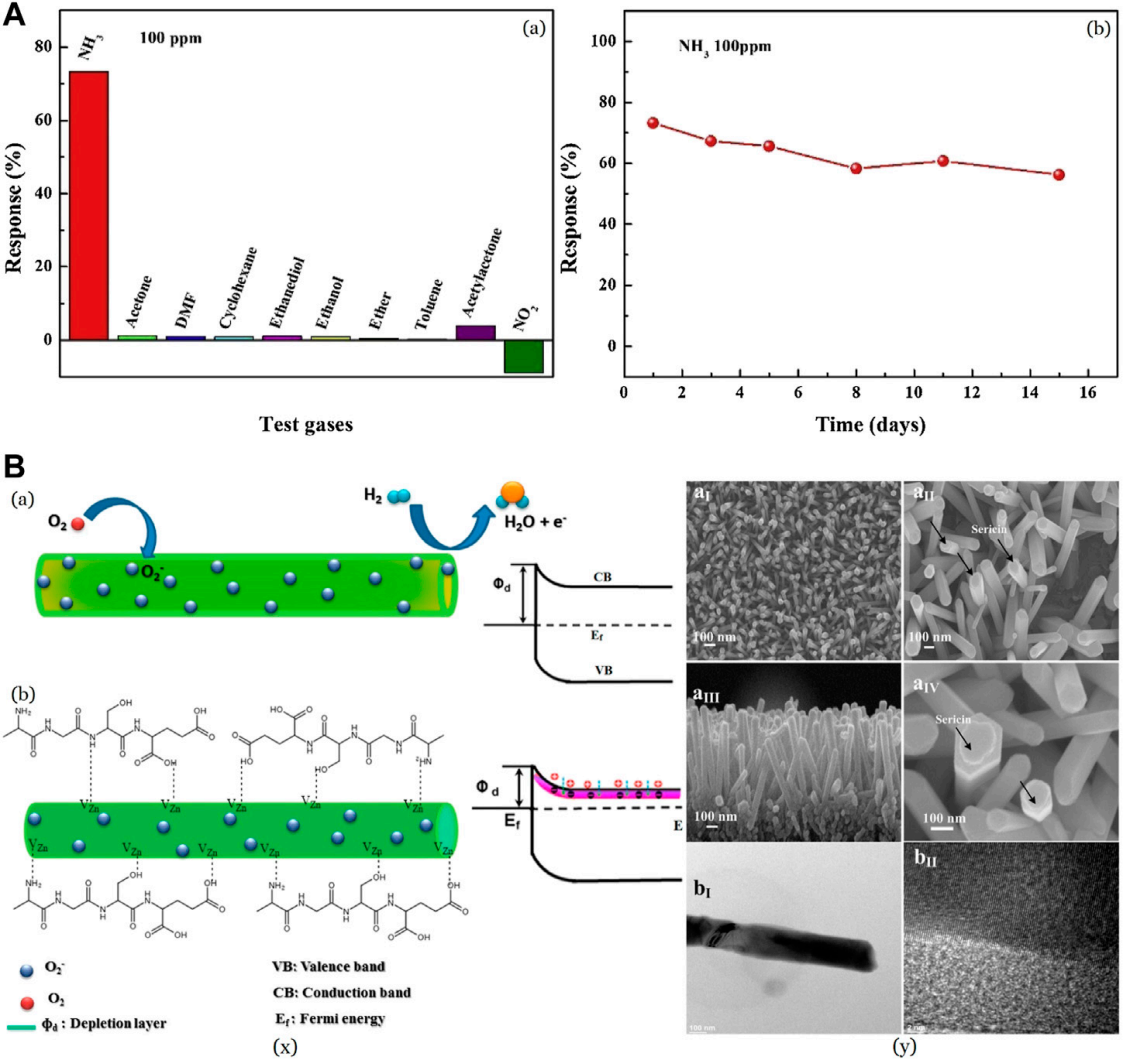
**(A)** Selectivity of the PPy/NS@silk-fiber sensor towards different test gases at the same concentration Reprinted with permission from (She et al., 2021). Copyright 2021 Elsevier **(B)** (x) Representational energy band diagrams in a H_2_ sensor in (a) pristine ZNR and (b) sericin-coated ZNR (y) (a) FESEM (b) HRTEM images showing the presence of sericin on ZNR Reprinted with permission from (Chuang et al., 2017). Copyright 2017 American Chemical Society.

The traditional sensor-supporting materials, such as Si (Pal and Jacob, 2004; Chartuprayoon et al., 2010; Hu, 2021), Interdigital Electrodes (IDEs) (Mazlan et al., 2017; Tang et al., 2020), Indium Tin Oxide and glass (Tiwari et al., 2015; Shankar et al., 2022) are limited in some applications which have terrible flexibility and adaption to complex environments. To achieve good flexibility, various substrates emerged over time, which include polyethylene terephthalate (PET), polydimethylsiloxane (PDMS), polyester (PE), polyimide (PI), and polycarbonate (PC). Silk fibroin is exploited as a possible flexible substrate. She et al. (2021) fabricated flexible PPy@silk-fiber and PPy@sponge sensors for detecting ammonia with silica nanospheres (NS) as a template while silk and sponge acting as a substrate in a facile *insitu* chemical oxidation polymerization method. Degummed silk is mainly used due to its ordered nature compared to raw silk with sericin. The PPy/NS@silk-fiber sensor possessed a lower base resistance, with a response of 2–5 times better than that of the PPy/NS@sponge sensor for 1–225 ppm NH_3_ at room temperature. PPy/NS@sponge sensor failed in detecting NH_3_ with less than 5 ppm concentration, while PPy/NS@silk-fiber gave a response. The resistance increased by 4% after 50 stretching cycles, while 200 cycles led to a decrease in response by 10.61% with hardly any change in response time. These results indicate that the PPy/NS@silk-fiber sensor possesses satisfactory mechanical stability for application as a wearable sensor. Several common VOCs, including acetone, N,N-Dimethylformamide (DMF), cyclohexane, ethanediol, ethanol, ether, toluene, acetylacetone, and NO_2_ were studied, and no considerable response was exhibited as shown in Figure 5A. Short response time (24 s) and recovery time (69 s) towards 100 ppm NH_3_ at ambient temperature indicating that the PPy/NS@silk-fiber sensor has good selectivity in differentiating NH_3_ from the concerned interfering gases and vapors.

The interaction between ammonia molecules and p-doped PPy is considered due to the compensation effect (Gustafsson et al., 1989). NH_3_ is an electron donating molecule, behaving as an n-type dopant, while the PPy layer acts as a p-type semiconductor (Bazzaoui et al., 2007; Šetka et al., 2017). Herein, adsorption of NH_3_ onto the PPy surface causes a decrease in the doping level, compensating for the effect of PPy. This induces electron transfer from NH_3_ molecules to the PPy main chains, causing a loss of the hole concentration and raising the electrical resistance (Joulazadeh and Navarchian, 2015; She et al., 2021). This can be explained in terms of ionization potentials of NH_3_ and the polymer (Yoshino and Gu, 1986).

#### 3.2.4 Hydrogen (H_2_)

Hydrogen is a non-toxic gas with no health hazards, but high concentrations in a closed environment cause asphyxiation due to the absence of oxygen. It has very low ignition energy and a wide range of combustible air mixtures, making it a very dangerous gas (starting from concentrations as low as 4%). A minute spark can lead to ignition, burning with a nearly invisible flame and having no odor, making its sensing an important aspect at the site of its storage. Its strong reducing activity causes some metals to become brittle, which could also be dangerous due to loss of structural integrity.

Chuang et al. (2017) fabricated zinc oxide nanorods coated with sericin (S-ZNR) for the detection of H_2_ gas. The S-ZNR was fabricated from sericin solution using an economical solvothermal method. Figure 5B (x) shows energy band diagrams for the response of (a) pristine and (b) S-ZNR to hydrogen adsorption (Chuang et al., 2017). Increased oxygen vacancies in S-ZNR compared to ZNR is profitable as it enhances the electrostatic interaction between the analyte gas molecules and the surface of the nanorods (Zeng et al., 2009). 100 ppm of H_2_ gave a sensitivity of 17.8% in S-ZNR while that of as-grown ZNR showed just ∼6.8%. Removal of adsorbed oxygen by reduction of sericin surface and chemisorbed oxygen moieties by H_2_ molecules leads to the release of trapped electrons into the sample, causing an increase in conductance. As shown in the SEM Figure 5B (y), sericin molecules get coated on the ZNR surface by the formation of complex ionized electronic states *via* weak electrostatic force. This may cause a surge in the electrostatic interaction between the functional groups of sericin and ZnO, boosting the number of oxygen moieties on the ZNR surface and, thus, the electronic transportation for sensor applications. The response and recovery time was observed to be better in S-ZNR, along with better sensitivity.

#### 3.2.5 Methanol (CH_3_OH)

At low concentrations, methanol vapors can be digested by the human system and exhaled through breath or urine. Methanol is always present in the environment at low concentrations as a by-product of the fermentation of plants, and no harmful effects have been observed. But, at high concentrations, due to its low flash point, a spark or even hot surfaces would cause ignition. It also has adverse effects on plastics and rubber in the surrounding. Inhalation of methanol vapors (200–375 mg/L) causes dizziness, drowsiness, blurred vision, and nausea. Chronic exposure (800–3,000 mg/L) could result in temporary or permanent blindness. At high concentrations, it can lead to inebriation, causing a vegetative state and finally leading to death. It can remain in the atmosphere for 18 days before either dissolving in water or eventually breaking down. Gas sensors detecting methanol are very much important to workers who are exposed to methanol vapors or are in a high-risk environment surrounding methanol storage containers.

**FIGURE 6:**
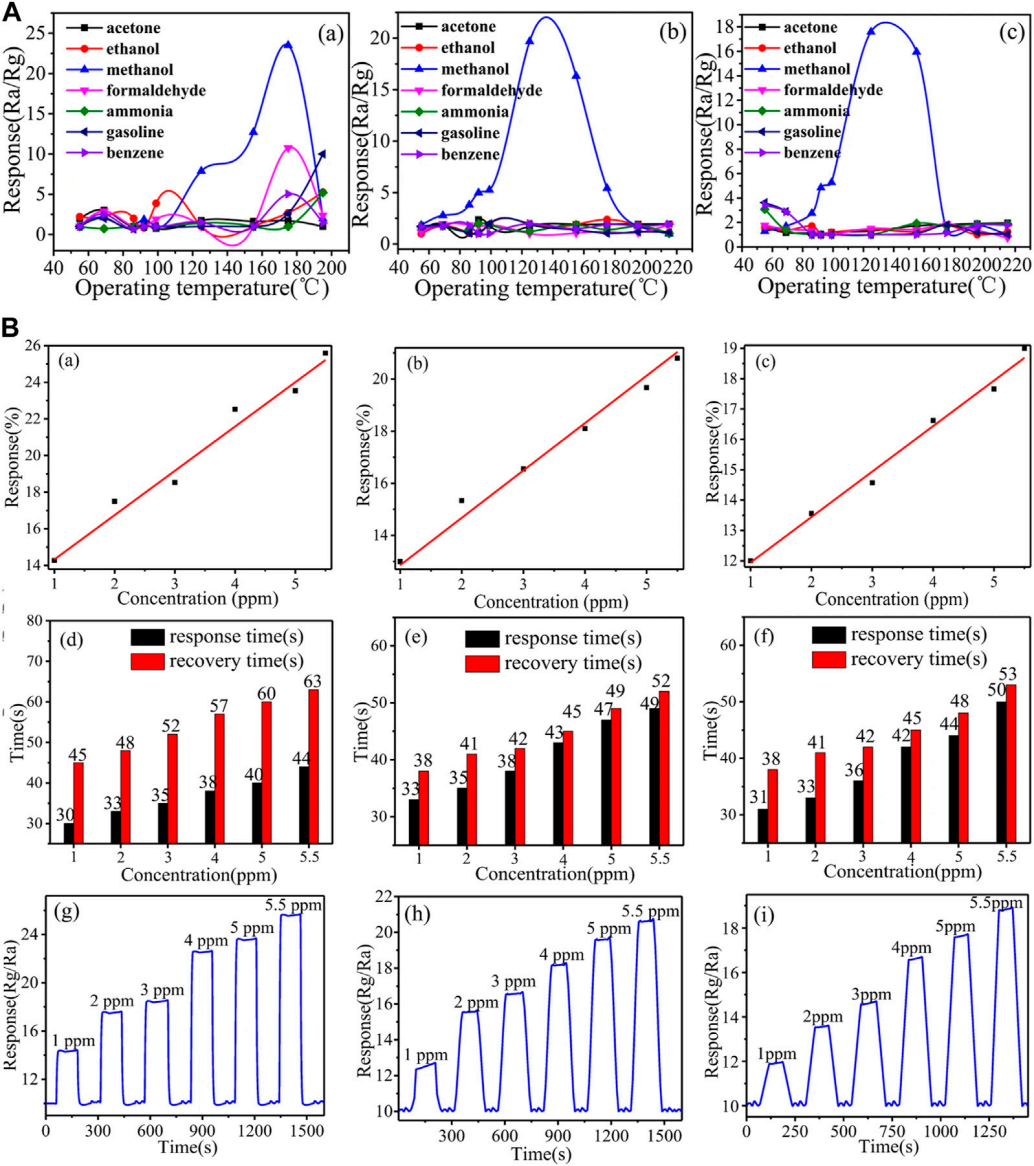
**(A)** Responses in ALMFs based gas sensors (a) ALMFs-1 (b) ALMFs-2 (c) ALMFs-3 towards various test gases at 5 ppm concentration **(B)** (a–c) Response% variation with concentration of methanol for ALMFs-1, 2, and 3, respectively (d–f) Response-Recovery time representation at various concentrations of CH_3_OH vapor (g–i) Dynamic response of this sensor at increasing concentrations of CH_3_OH Reprinted with permission from (Qian et al., 2017). Copyright 2017 Springer Nature.

Ag-LaFeO_3_ Molecular Imprinted Polymers (ALMIPs) fibers with recognition centers are investigated as highly selective methanol sensors (Qian et al., 2017). Good surface area, thermal stability, rich active oxygen lattice, structure tunability, and strong reducibility have unfolded Ag-LaFeO_3_ (AL) as a potential material for gas sensing (Traversa et al., 1995). Molecularly Imprinted Technology (MIT) is used to produce polymers that provide the active binding site for the analyte molecules by matching space structures. These host polymers show high molecular recognition of the selected template molecule. Briefly, a sol of AL was prepared using their elemental nitrates, [AgNO_3_, LaNO_3_.6H_2_O, Fe(NO_3_)_3_.9H_2_O] and mixed with citric acid and polyethylene glycol and then used as cross-linker in the MIT. Methacrylate (MAA) was used as a functional monomer, AL was used for cross-linking, and methanol was used as a molecular template for recognition sites to fabricate a highly selective methanol analyte-sensing material. Different templates like silk, filter paper, and carbon fibers were used to obtain high surface areas and analyzed for gas sensing. These templates were burnt out in the air at 800°C for 2 h to obtain ALMIPs fibers and ALMFs. Various templates make fibers acquire distinct exposed facets (Gao et al., 2008; Tang and Wang, 2015b), and various neighboring lattice has an impact on obtaining diverse properties. The surface areas of ALMFs were in the order filter paper (ALMFs-1) > silk (ALMFs-2) > carbon (ALMFs-3). ALMFs-1 showed 23.5% (at 175°C) response while ALMFs-2 showed 19.67% (at 175°C) and ALMFs-3 showed 17.59% (at 125°C). Here ALMFs were also investigated for other organic vapors, formaldehyde, acetone, ethanol, ammonia, gasoline, and benzene. ALMFs-1, ALMFs-2 and ALMFs-3 have shown lower responses <10, <3, and <2 respectively for organic vapours shown in Figure 6A. Also, the response relationship at various amounts of CH_3_OH, response recovery time, and dynamic response is shown in Figure 6B.

ALMFs sensing mechanism is similar to LaFeO_3_, which is a typical p-type semiconductor, exhibiting variation in resistance prior to and after exposure to the analyte gas (Haupt, 2011). Calcining at elevated temperatures creates lanthanum vacancies at the crank points of the cell, and the resistance variation is seen as a result of ionization of lanthanum vacancies (Mizusaki et al., 1983; Liu et al., 2011). O_2_ gets adsorbed on LaFeO_3_ surface on exposure to air and captures free electrons of the LaFeO_3_ particles as a result of greater oxygen electronegativity forming chemisorbed oxygen ionic species resulting in an increase in the number of positive charge carriers (holes) in the valence band and increase in conductivity of materials due to higher available carriers (Niu et al., 2004). CH_3_OH reacts with the ionized oxygen species on the material to form Carbon dioxide and water, releasing the trapped electrons and causing a widening of the space-charge layer leading to the increased potential barrier and hence, the resistance.

#### 3.2.6 Humidity

Moisture is a vital moiety of our environment with significant influence on living and non-living matter. This leads to necessary regulations in a multitude of manufacturing industries and for improvement of quality of life for humans in fields such as control of living environments in buildings, in hospitals to monitor respiratory equipment, in agricultural irrigation to control the amount of H_2_O to name a few. Relative Humidity (RH) of air can be measured by humidity sensors, which use materials to sense the concentration of water vapor in air or pure gas and display it (Rajkumar and Kumar, 2019).

Sensing humidity exploits the water adsorption and desorption process of a few materials. The commonly used measurement units in humidity sensors are Relative Humidity (RH), Dew/Frost Point (D/F PT), and Parts Per Million (PPM). RH is the ratio of the partial pressure of water vapor present in a gas to the saturation vapor pressure of the gas at a given temperature, making it a function of temperature (thus relative). It is a unitless quantity and written as a percentage value. The dew point is the temperature below which the water vapor in a gas starts condensing into liquid. At dew point, the RH is deemed to be 100%. Similarly, the frost point is the temperature at which the vapor begins to freeze over ice or exposed cold surface. D/F PT is an absolute humidity unit. PPM is the water vapor concentration in mg/L of air or gas. PPM, similar to D/F PT, is also an absolute measurement. Although this unit can be hard to comprehend, it is used extensively in industries for trace measurements (Chen and Lu, 2005).

**FIGURE 7:**
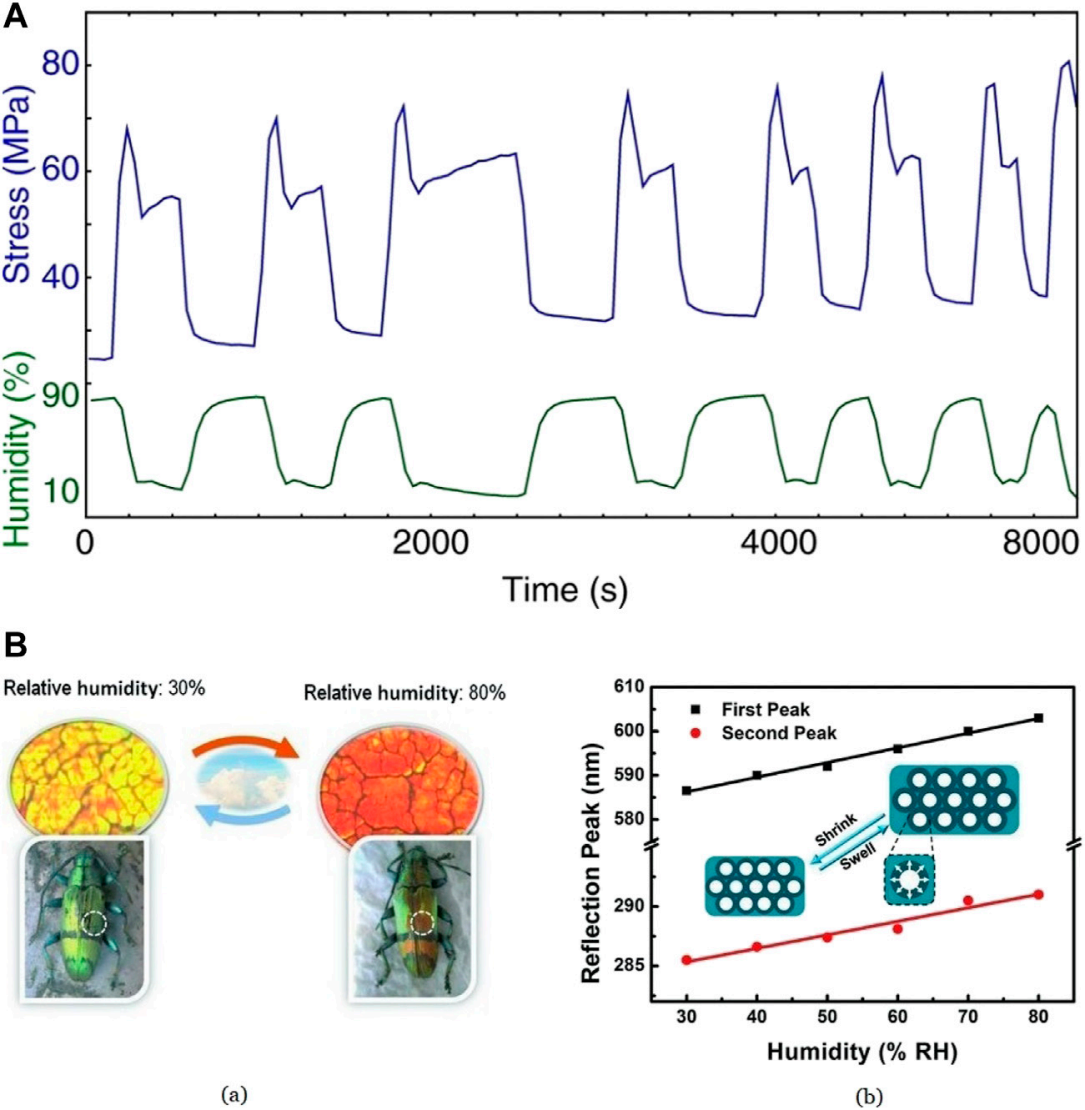
**(A)** Time vs. Stress curve for differing values of humidity Reprinted with permission from (Agnarsson et al., 2009). Copyright 2009 The Company of Biologists **(B)** (a) The color of Silk PCs characterized through optical microscopy shows a transition from orange at 80% RH to yellow at 30% RH, showing the biomimicking beetle’s humidity response (b) Proportional relationship between RH value and reflection peaks Reprinted with permission from (Diao et al., 2013). Copyright 2013 John Wiley and Sons.

Silk fibroin (SF) inverse opals were considered for the synthesis of humidity-responsive photonic crystals (Diao et al., 2013). The cyclic contraction property of silk fibroins due to changes in humidity and the ability to alter the structural parameters of inverse silk opals make them the best material for this application (Agnarsson et al., 2009; Fu et al., 2009). Briefly, a 2% w/v aqueous solution of SF was prepared (Sah and Pramanik, 2010) and fabricated into inverse opals using 3D colloidal crystal templates and then coated with polystyrene to obtain Silk Photonic Crystals (PCs). A high RH causes the adsorption of water to the irregular coils in the amorphous region of molecules in silk fibers, causing a disruption of the weak hydrogen bonds. This induces a drop in the stress–strain curve due to the relaxation of silk, as shown in Figure 7A (Agnarsson et al., 2009). On drying, H_2_O molecules evaporate from the irregular coil region, reforming the hydrogen bonds and causing immobilization of silk molecules, contracting the fibers. After 6,500 s or 3 rounds of repetitions of the tension, not much variation was observed in the measurements, making it a suitable candidate for precise control of their optical property. It was observed that at a high RH value, the visible 350 nm reflection peak undergoes a redshift due to the swelling of SF shells by water adsorption causing changes in its refractive index. However, the reflection peak of the SF inverse opal shows a blueshift (towards a lower wavelength) with a decrease in the RH level. The sensor was shown to be responsive between 30% and 80% RH. Figure 7B (Diao et al., 2013) shows the color change of PCs to change in RH and the dependence of reflection peak on humidity. The elastic nature of SF (Nakamae et al., 1989) keeps the inverse opals crack-free.

**FIGURE 8:**
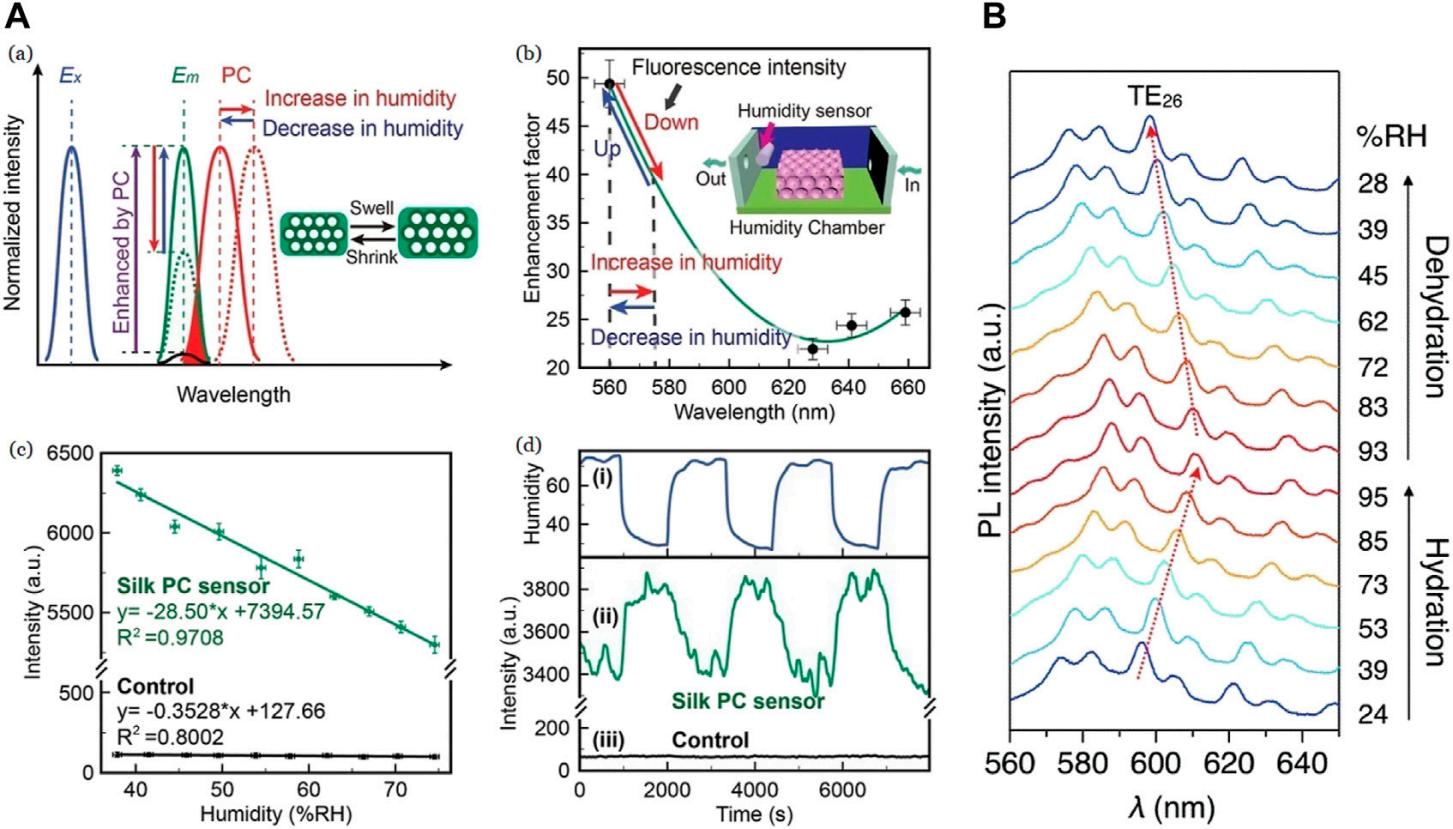
**(A)** (a) Plot of fluorescence intensity to show Photonic Band-Gap shift (red) as a result of humidity variations. Optimized photonic crystal structure (“E_x_”—blue) and silk photonic crystals (“E_m_”—green) improved intensity multiple times for same incident light compared to that of the control (black). Inset: Changes in silk fibroin shells (b) Trend forecast of the fluorescence intensity of silk photonic crystal with varied stopbands. The enhancement factor indicates the ratio of fluorescence intensity of silk photonic crystal films to that of the control. The stopbands of silk photonic crystal films are situated at 560, 628, 641, and 659 nm. Inset: representation of a humidity-controlled chamber (c) Fluorescence emission at varied humidity levels compared between pristine fluorescent silk film (black) and PC fluorescent silk film (green) with 1 s integral time d) Fluorescence measurement with integral time of 0.5 s of the biocompatible humidity sensor with rapid variations in humidity. (i) RH value compared with fluorescence shifts in (ii) PC fluorescent silk (green) and (iii) pristine fluorescent silk film (black) Reprinted with permission from (Fan et al., 2019). Copyright 2019 John Wiley and Sons **(B)** Photo-luminescence spectra showing humidity-dependence of an excited single SF microsphere with a continuous wave laser (*λ*_ex_ = 450 nm) Reprinted with permission from (Heah et al., 2021). Copyright 2021 Royal Society of Chemistry.

Fan et al. (2019) fabricated a similar humidity sensor making use of the excellent optical property of SF where Rhodamine 6G (R6G) was doped on silk, coupling its fluorescence emission with the inverse opal PC. The silk PC films are highlighted to transform into a “self-collimator” by enhancing fluorescence emission or by improving fluorescence detection. The R6G moieties were evenly distributed throughout SF films due to hydrogen bonding between silk fibroin and Rhodamine 6G molecules. This interaction happens in the mesoscopic scale (Lin et al., 2016). The sensing activity was measured from 37% to 74% RH and showed a sensitivity of 28.5%. Similar to the previous sensor, humidity variation caused cyclic contractions in SF, leading to the changes in the lattice parameter value (Agnarsson et al., 2009). The SF inflammation due to high RH causes the expansion of silk shells, leading to a redshift (towards higher *λ*) in the reflection peak. Contrarily, when the RH decreases, the silk shells contracted due to SF deflation leading to an increase of fluorescence emission of the silk PC due to blueshift (towards higher *λ*) in the Photonic Band-Gap (PBG) as seen in Figure 8A (Fan et al., 2019).

The optical property of SF (Perry et al., 2008; Bucciarelli et al., 2018) was also used for the fabrication of optical humidity sensors (Heah et al., 2021), where absorption of a large volume of water vapor causes a slight variation in the refractive index and optical path length, maintaining comprehensive morphology and optical connection of the device. A modified mini-emulsion method was used for SF microspheres fabrication and then stained with AR52 to harness its photoluminescence (PL) property. DMF is used to swell the microspheres for easy diffusion of dye into them. The doped microsphere has a pale red appearance and emits orange–red PL. The humidity sensor has been tested for the values of 24%–95% RH, and a sensitivity of 187 pm/% was observed. The effect of humidity was studied using *μ*-PL spectroscopy experiments. When RH increased, the resonant peaks were observed to recede toward a longer wavelength while advancing toward a shorter wavelength when RH was lowered. The microspheres absorbed moisture leading to radius enlargement, which is reflected in the peak shift shown in Figure 8B. This microsphere is claimed to respond to the humidity change within 1 min, surpassing a commercial humidity sensor that took 5 min to reach a stable state.

**FIGURE 9:**
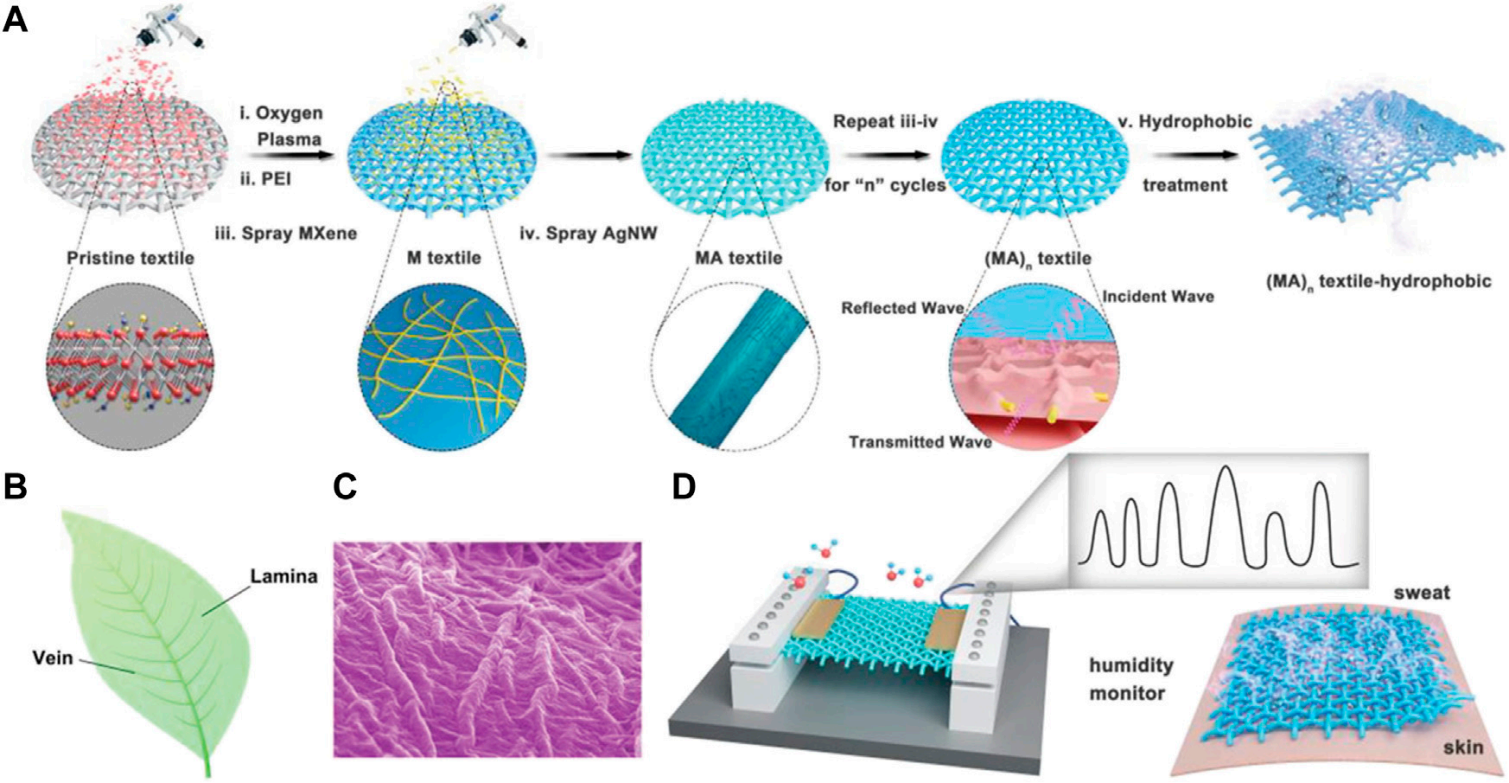
**(A)** Illustration showing approach to fabrication **(B)** Leaf picture **(C)** biomimicking leaf network with 2D MXene nano-sheets and 1D Ag-NWs on silk substrate **(D)** Fabricated sensor sensing human sweating Reprinted with permission from (Liu et al., 2019). Copyright 2019 John Wiley and Sons.

A resistive humidity sensor was assembled by Liu et al. (2019) through a vacuum-assisted layer-by-layer assembly approach. Silver 1D Nanowires (Ag-NWs) and MXene 2D nanosheets have been incorporated into SF, producing high conductivity, bio-mimetic, and leaf-like nanomaterials as seen in Figure 9 (Liu et al., 2019), exhibiting intriguing properties like highly sensitive RH response, EMI shielding, and superhydrophobicity. SF was given an oxygen plasma treatment, followed by soaking in polyethyleneimine to add polar groups for efficient loading of MXenes and Ag-NWs alternatively to produce a highly conductive network. This method of preparation ensures the retention of permeability of textile substrate and its porosity.

When water molecules get adsorbed between the interlayers, MXenes exhibit hydration/dehydration behavior due to the surface interaction and charge transfer processes (Ghidiu et al., 2016; EricMuckley et al., 2017; Muckley et al., 2018). This behavior and the presence of pores propel this material for potential humidity sensor application. The coated silk showed stable and reproducible resistance changes within the cyclic testing in a range of RH (∼0%–85%), indicating stability and durability to moisture, possibly as a result of the superhydrophobicity preventing the potential degradation of MXenes due to accumulation of water. The sensor featured a response time of 5 s and a recovery time of 80 s, which is comparable to a commercial humidity sensor.

**FIGURE 10:**
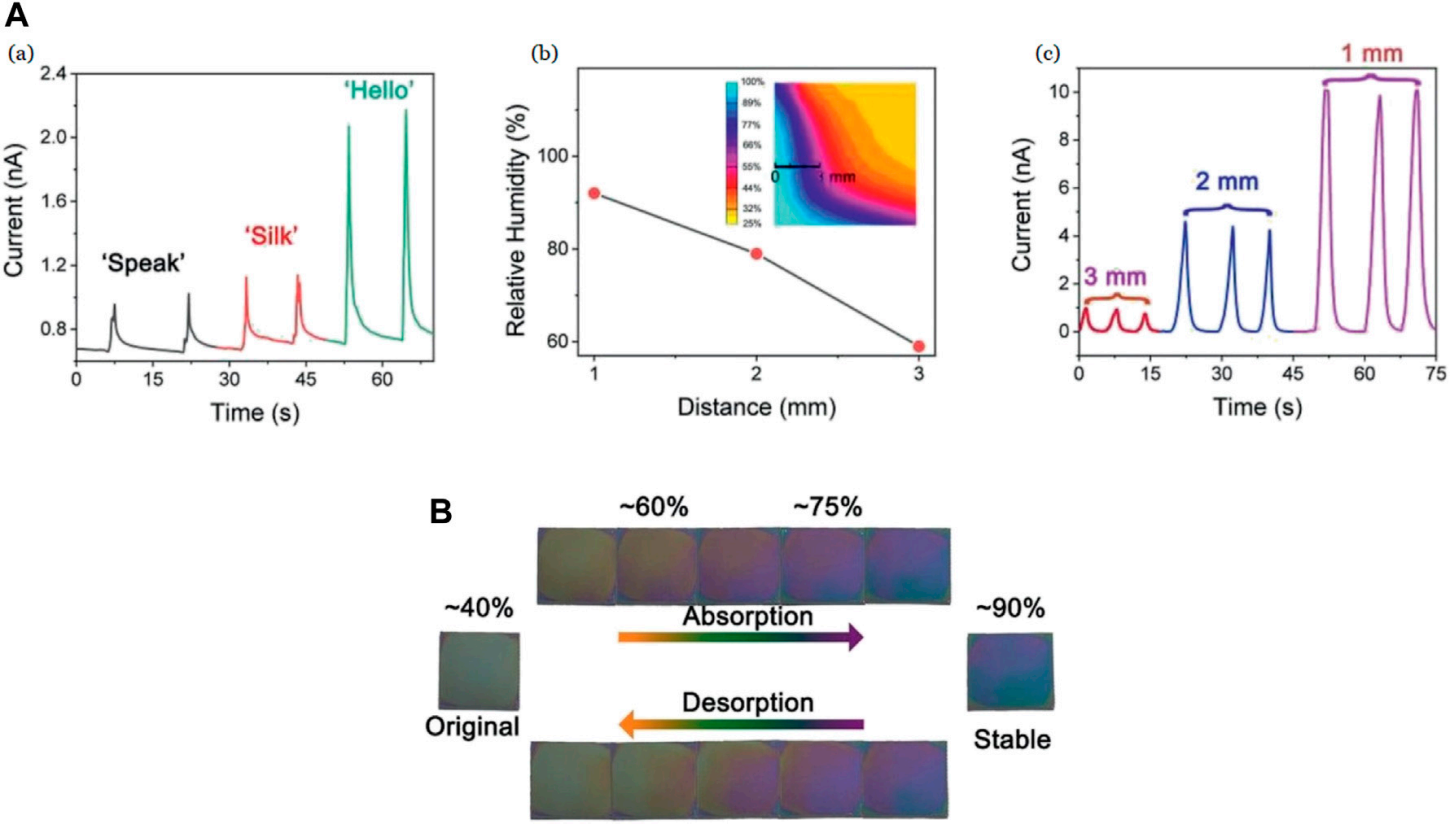
**(A)** (a) Current variation of humidity sensor against time for different words, such as “Speak,” “Silk,” and “Hello.” (b) Relative humidity of fingertip change with respect to distance. (c) Recurrent response curves of the non-contact sensor (1 mm, 2 mm and 3 mm) **(B)** Color change between humidity levels of 40%–90% Reprinted with permission from (Zheng et al., 2021). Copyright 2021 John Wiley and Sons.

Zheng et al. (2021) have used SF as an active sensing material for humidity sensors with non-contact sensing action, and high sensitivity. An aqueous solution of degummed SF (Sah and Pramanik, 2010) was deposited on a PET substrate with interdigitated Ag electrodes. With an increase in the RH, the sensor shows an increase in the current with the amount of gain relative to the RH percentage. The response/recovery duration interval of the sensor is shown to be 100 s with a sensitivity of ≈750% at 85% RH while it was characterized at RH values between 43% and 95%. The sensor is also depicted to differentiate spoken syllables associated with the moisture changes in speech. In the non-contact sensing action, the moisture around the fingertips was captured and led to an increase in the current with a decrease in the distance between the fingertip and the sensor, both of which are shown in Figure 10A (Zheng et al., 2021). The SF films were also observed to change color in response to changes in RH from pale yellow to blue [Figure 10B (Zheng et al., 2021)]. These features show the possible applications in human activity identification, anti-counterfeiting, human–machine interactions, and optical humidity sensors.

Composites of SF with rGO have been prepared to improve the electrical conductance of the silk materials for their operation in the biosensors field. Zhang et al. (2019) have composited SF with graphene to fabricate flexible and wearable electronics with good skin comfort. Aqueous silk solution (Sah and Pramanik, 2010) was mixed with butanediol, followed by a uniform mixture with a dispersion of graphene and PVA (PolyVinyl Alcohol), and dropped on a PDMS template. Water content had an influence on the mass swelling rate, which caused a proportional change in the sheet resistance of the film. When the RH value was increased from 0% to 100%, the resistance was observed to increase by 200 times. In both wet and dry conditions, great repeatability and excellent mechanical properties were observed, and this has been proposed for use in biomedical materials, wearable sensors, and implantable internal sensors.

Using SF as the substrate, nickel interdigital electrodes were prepared by electroless coating (Jiang et al., 2008) and GO_x_ suspension was spray-coated over it to produce humidity sensing layers (Li et al., 2018). Integration into a face mask of a healthy volunteer for real-time tracking of respiratory profiles was carried out. The increase in humidity leads to an enhancement in the current due to a decline in the resistance and *vice versa*. The differences between deep and fast breathing were easily differentiated due to different humidity production. The device was also shown to endure 2,500 recurrences of bending and twisting without affecting its performance, and repeated usage of the device did not have much influence on the sensor. Thus, a respiration-sensing device based on SF is proposed for application prospects in monitoring the basic human health status.

**FIGURE 11:**
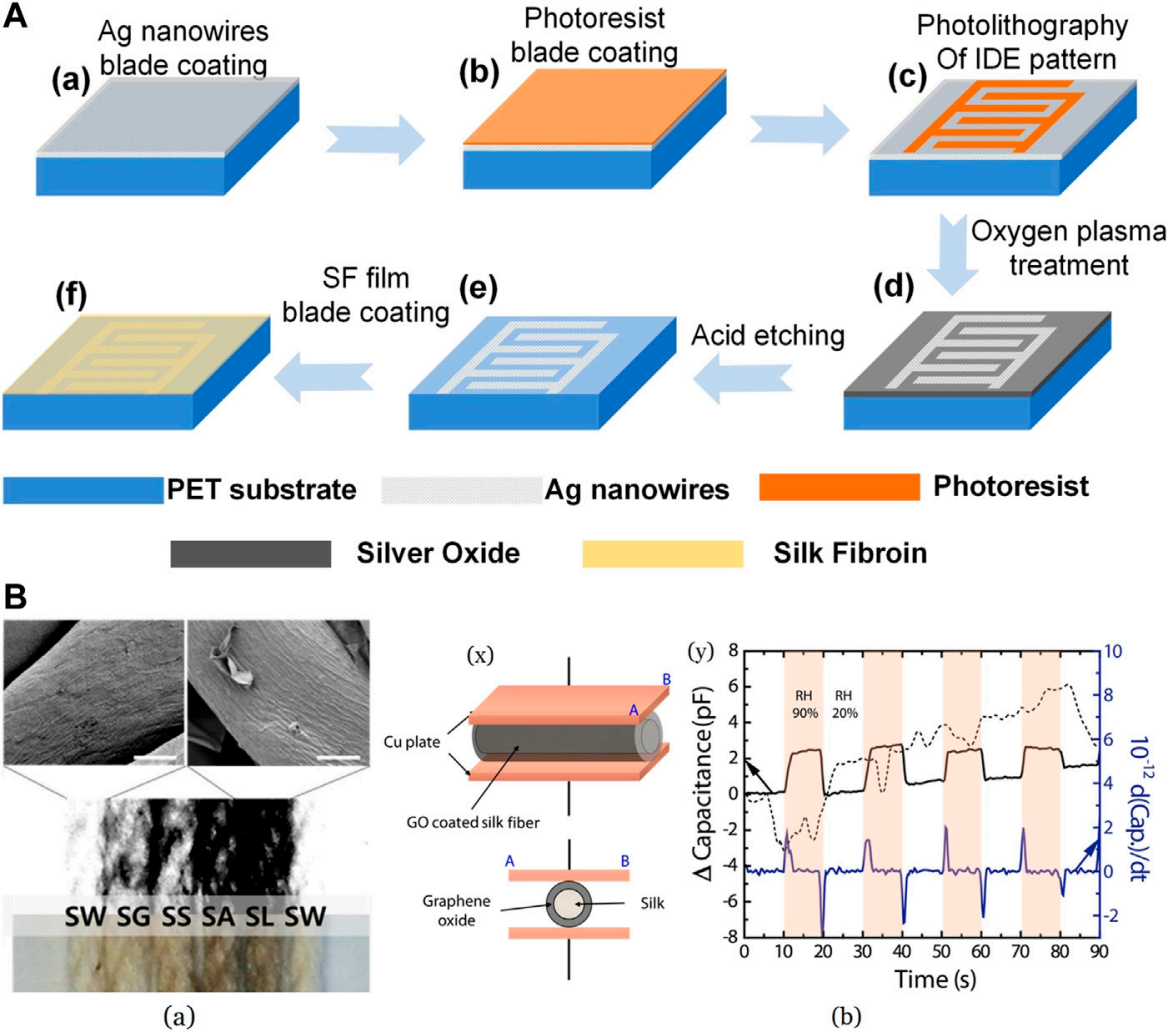
**(A)** Silk fibroin film-based humidity sensor fabrication procedure Reprinted with permission from (Luo et al., 2020). Copyright 2020 Elsevier **(B)** (a) Different GO_x_ coated Silk fibroin, and Scanning Electron Microscopy image of SW and SL (scale—10 *μ*m) SW—SF submerged in DI water only; SF rubbed with a glass bar—SG, nothing (control)—SS, aluminum foil—SA, and latex gloves—SL (b) (x) Representative picture of capacitive humidity sensor where GO_x_ coated SF was used (y) The capacitance and derivative capacitance curve of sensor for 20%–90% RH values Reprinted with permission from (In Han et al., 2017). Copyright 2017 MDPI.

Silk fibroins have been explored for capacitive humidity measurements as well. SF is a dielectric material with outstanding electric field insulation and tends to vary its dielectric constants with changes in humidity hence the capacitance. SF has alternating hydrophobic crystallite regions, and hydrophilic amorphous blocks (Taketani et al., 2005). An aqueous solution of SF was blade-coated on an interdigital electrode of Ag-NWs and SU8 photoresist blade-coated over PET substrate shown in Figure 11A (Luo et al., 2020). A higher proportion of hydrophobic *β*-sheets is preferred as it prefers the water adsorbed to be unbound without breaking any H-bonds. This leads to a better response rate as it escapes easily. In the case of random coils, bound water is formed due to the evolution of H-bonds between water and polymer. This brings about an increase in response time, which is not beneficial. By sticking a sensor on the skin between the nose and lips of a volunteer, breathing assessment tests were carried out. The capacitance readings were stable when measured in the same mode, and the variations in the breathing resulted in noticeable changes. The variation in the capacitance value was also measured between 40% and 95% RH values with excellent sensitivity. This method of fabrication was proposed to be economical and durable without any degradation due to moisture.

SF coated with GO_x_ materials was investigated for wearable, flexible applications, where quantity and quality of the GO_x_ were regulated by variation in electrification force on silk fibroins (In Han et al., 2017). Also of interest was the adhesive-free coating of GO_x_ on silk. SF was rubbed against a few test materials to control the amount of positive charge accumulation on silk and then coated with GO dispersion, which contains negatively charged functional groups. Latex gloves-rubbed SF carried the maximum charge leading to the best coating shown in Figure 11B (a) (In Han et al., 2017), giving it maximum conductivity as well. This material was used for sensor fabrication by embedding it between two copper plates as seen in Figure 11B (b) (In Han et al., 2017) for capacitive measurements. Good response-recovery behavior with high repeatability in the RH range of 20%–90% at ambient temperature was noticed in the sensor. Van der Waals, forces between oxygen functional groups of GO_x_ and H_2_O, is ascribed as the reason for remarkable sensing capability. Authors have anticipated that this approach will facilitate the manufacture of flexible, low-energy consumption devices at cheaper rates.

**FIGURE 12:**
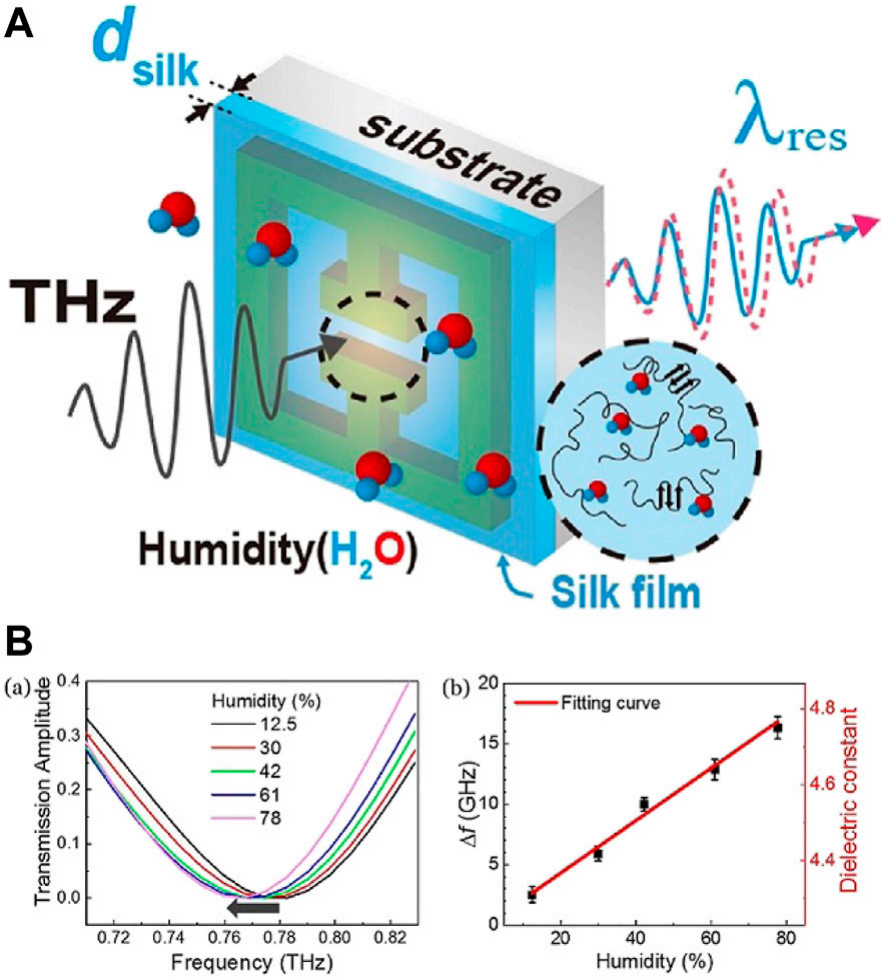
**(A)** Illustration of the hybrid THz humidity sensors. The SF film was coated on the THz metamaterials **(B)** (a) Transmission amplitude for various RH between 12.5%–78% (b) Frequency shift dependence on humidity, obtained from (a) Reprinted with permission from (Kim et al., 2018). Copyright 2018 Optica Publishing Group.

A humidity sensor was also made by incorporating silk into metamaterials operating at THz frequency and detecting humidity based on the change in resonant frequency due to change in permittivity of gap area (Park et al., 2014; Park et al., 2016), which is occupied by silk fibroin (Kim et al., 2018). The aqueous solution of SF (Sah and Pramanik, 2010) was coated over a THz metamaterial made from Cr/Au (Figure 12A). Metamaterials show a gap structure that induces capacitive nature by charge aggregation when a circular current is produced by the incident wave leading to LC (Inductive-Capacitive) resonance. Changes in the permittivity of the silk due to the effect of humidity change the LC resonance, thus modifying the resonant frequency. As RH increases, the resonant frequency shifts toward red, indicating an increase in dielectric constant (Figure 12B). Humidity showed no deterioration in the silk film over the course of measurements. When water is absorbed into silk, hydrogen bonds between SF proteins are broken. However, water evaporation shows no variation in the molecular structure of the proteins. The sensor measurements were performed for RH values of 12.5%–78% with a variation of 2.3% in RH detectable having a response% of 0.22 GHz/%. The film thickness regulates the recovery time, with a display of a recovery time of 10 s. Further research is needed to optimize the devices by introducing an improved metamaterial with enhanced-Q factors, high-speed THz measurement tools, and potential by modifying the chemical properties of SF.

## 4 Outlook

In the literature discussed here, most groups have used SF as a substrate rather than an active material. Metal oxides/polymers, which are already widely considered sensor materials, are coated on the SF material. The main application one can see the connectivity imparted by SF as a substrate in gas sensors or as a gel that binds different materials in a hybrid while imparting changes in properties on the metal oxide semiconductors. The different conductivity ranges that a carbonized SF can impart on the hybrid material is to be exploited. Even though many carbon materials (e.g., carbon nanotubes, and graphene) can be used for conductivity and connectivity reasons but they are cost-ineffective, also functionalization is needed prior to hybridization. SF has highly repeated amide functional groups, intermolecular hydrogen bonds, and are assembled due to van der Waals interactions. These structural properties help in anchoring them to the polymers and metal oxides without any severe treatments. These SF can also be dissolved and used in solution form for obtaining printed film sensors through soft lithography, contact printing, inkjet printing, spin coating, stenciling, and nano imprinting if not directly as substrates.

Even with these advantages and its easy availability, silk has not been explored for active gas sensing materials. A few paths that researchers can look into are the coating of different transitional metal oxides on the fibroin substrate, controlling the carbonization levels of silk for tuning their electrical properties, and using the dielectric property of these highly functional materials in sensing. The changes in the dielectric and optical properties of the fibroin films are explored for the humidity sensors. Further tuning of these sensors can be done by the introduction of metal nanoclusters, nanoparticles to control the photo-luminescent properties. Upon interaction analyte gas molecules with these hybrids shows characteristic changes in luminescence properties which can be used for sensing applications. The inclusion of additives in the diet of the silkworms along with the mulberry leaves leads to desirable changes in the structure and properties of the silk material obtained, on which further research is required. Wireless passive antennas on silk substrates across multiple regions (MHz, GHz, THz) of the electromagnetic spectrum are one concept that can detect the changes in resonant responses on interaction with analyte gas. The very important advantages of silk commercialization are their easy processability in device fabrication, large-scale silk production with minimum lab facilities, and semi-skilled labor.

## 5 Conclusion

We have reviewed the processing of SF and its structural properties for an investigation into their effect on gas sensing applications. The different types of sensors based on their working principles have also been briefed. Silk-based sensors have been shown to be applied for sensing different gases, with each of them being selective. The synergistic effect of silk and metal oxides towards selective sensing application has been observed in the case of ZnO, SnO_2_. SF has also been used as a template in the case of LaFeO_3_ sensor. Sensors of polymers have been fabricated with SF as the substrate. They show good response and selectivity due to SF’s electronic properties leading to better response to gas adsorption. Humidity sensors fabricated using silk have also been designed on the principle of shifting the photoluminescent peak due to changes in silk structure on interaction with humidity. It was observed to have a redshift at a higher RH and a blue shift at a lower value. It has also been shown to change the resistance and capacitance values during sensing, as seen in the devices. It has also been incorporated into metamaterials to exploit its behavior on exposure to humidity. As observed, SF has been applied in sensor fabrication as both the substrate and the active material. The porosity of SF also provides cave-like space for optimum gas adsorption. There is scope for exploring the different 0D (metal oxides), 1D (nanowires/rods), and 2D (inorganic graphene analogs)-SF sensors for industrial sensor fabrications and the incorporation of sericin for sensor devices.
